# PEGylated Elesclomol@Cu(Ⅱ)-based Metal‒organic framework with effective nanozyme performance and cuproptosis induction efficacy for enhanced PD-L1-based immunotherapy

**DOI:** 10.1016/j.mtbio.2024.101317

**Published:** 2024-10-28

**Authors:** Xufeng Lu, Wenhai Deng, Shuaibin Wang, Shengsheng Zhao, Bingzi Zhu, Binglong Bai, Yiwen Mao, Ji Lin, Yongdong Yi, Zuoliang Xie, Xiang Wang, Yongyong Lu, Xiufeng Huang, Tao You, Xiaolei Chen, Weijian Sun, Xian Shen

**Affiliations:** a Zhejiang International Scientific and Technological Cooperation Base of Translational Cancer Research, Department of Gastrointestinal Surgery, The Second Affiliated Hospital and Yuying Children's Hospital of Wenzhou Medical University, Wenzhou, Zhejiang, 325000, China; b Zhejiang Key Laboratory of Intelligent Cancer Biomarker Discovery and Translation, Department of Gastrointestinal Surgery, The First Affiliated Hospital, Wenzhou Medical University, Wenzhou, Zhejiang, 325000, China; c Research Center of Basic Medicine, The Second Affiliated Hospital and Yuying Children's Hospital of Wenzhou Medical University, Wenzhou, Zhejiang, 325000, China; d Oujiang Laboratory (Zhejiang Lab for Regenerative Medicine, Vision, and Brain Health), School of Laboratory Medicine and Life Sciences, Wenzhou Medical University, Wenzhou, Zhejiang, 325000, China; e Department of Breast Surgery, The Second Affiliated Hospital and Yuying Children's Hospital of Wenzhou Medical University, Wenzhou, Zhejiang, 325000, China; f Department of Urology, The First Affiliated Hospital of Wenzhou Medical University, Wenzhou, Zhejiang, 325000, China

**Keywords:** Cu(Ⅱ)-MOF, Nanozyme, Cuproptosis, Breast cancer, Anti-PD-L1 antibody, Immunotherapy

## Abstract

Nanozymes constitute a promising treatment strategy for antitumor therapy. However, the catalytic function of metal‒organic framework (MOF)-based nanozymes during cuproptosis remains unclear. In this study, a Cu(Ⅱ)-based MOF nanocomposite loaded with the copper ionophore elesclomol and surface modified with polyethylene glycol polymer (PEG) was developed (ES@Cu(Ⅱ)-MOF) for effective cuproptosis induction. The peroxidase (POD)-like activity of ES@Cu(Ⅱ)-MOF generated an abundance of hydroxyl radicals (•OH) via a Fenton-like reaction, and its glutathione peroxidase (GSH-Px)-like activity converted Cu^2+^ into more toxic Cu^+^ ions to efficiently consume endogenous GSH. Notably, the rapid accumulation of Cu^+^ and ES in tumor cells induced the aggregation of lipoylated dihydrolipoamide S-acetyltransferase (DLAT) and the downregulation of Fe‒S cluster proteins, ultimately leading to cuproptosis. ES@Cu(Ⅱ)-MOF exhibited extraordinary cytotoxicity against breast cancer cells *in vitro* and significantly suppressed 4T1 breast tumor growth *in vivo*. Moreover, ES@Cu(Ⅱ)-MOF induced immunogenic cell death (ICD) to increase the antitumor immune response. Furthermore, combining ES@Cu(Ⅱ)-MOF with an anti-programmed cell death-ligand 1 (PD-L1) antibody converted the immunosuppressive tumor microenvironment to an immunogenic microenvironment, thus effectively inhibiting breast tumor growth. Overall, this work provides an innovative approach utilizing nanozymes to facilitate cuproptosis for cancer treatment, which potentially enhances the effectiveness of immune checkpoint inhibitor-based immunotherapy.

## Graphical abstract

**Figure:**
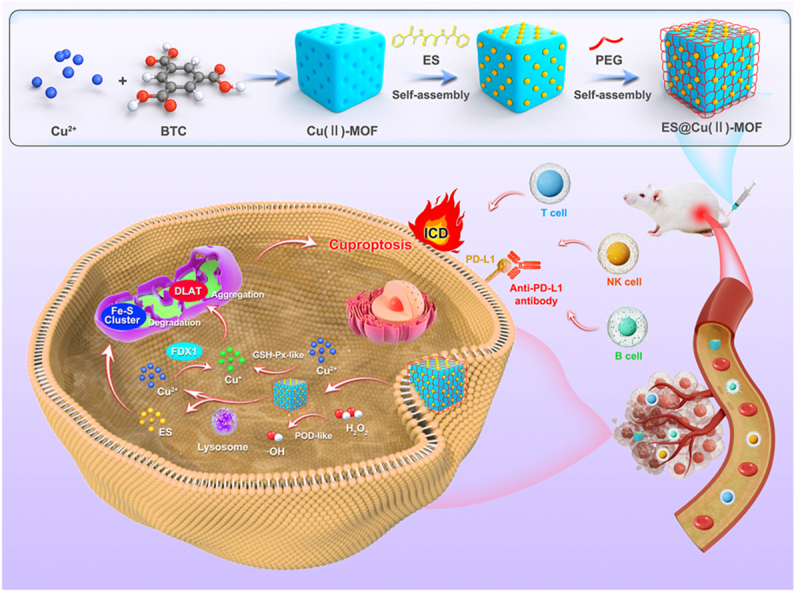

## 1 Introduction

Cuproptosis, a novel form of programmed cell death, is different from traditional forms of cell death, such as apoptosis, necroptosis, pyroptosis, and ferroptosis [1]. Cuproptosis is precipitated by excessive intracellular accumulation of copper ions (Cu^2+^), which leads to the aggregation of lipoylated proteins, particularly dihydrolipoamide S-acetyltransferase (DLAT), within the mitochondrial tricarboxylic acid (TCA) cycle, leading to proteotoxic stress and ultimately to cell death [[2], [3], [4]]. Cuproptosis induction is a potential therapeutic strategy for cancers that are resistant to traditional treatments [5,6]. However, harnessing cuproptosis for cancer therapy presents challenges, such as the high level of intracellular copper required to induce the process and the potential for systemic toxicity owing to the nonselective accumulation of copper [7]. To address these issues, copper ionophores, such as elesclomol (ES), are used to facilitate the transport of Cu^2+^ into cells [8]. Additionally, the development of copper delivery systems using nanomaterials has shown promise in increasing the selectivity and accumulation of copper in tumor cells [9,10], thereby increasing the potential for cuproptosis induction. However, the application of cuproptosis-based therapies is in its infancy and requires further investigation.

Recently, nanozymes have become representatives of a groundbreaking class of nanomaterials that emulate the functions of natural enzymes, offering a novel and promising avenue for the advancement of therapeutics and diagnostics [[11], [12], [13]]. Nanozymes, such as catalase, peroxidase (POD), oxidase, and superoxide dismutase, which are pivotal in various biological processes and therapeutic interventions, have been engineered to exhibit a range of enzyme-like activities [14]. These synthetic catalysts not only replicate the catalytic activities of enzymes but also harness the unique properties of nanomaterials, such as a high surface area, tunable surface chemistry, and responsiveness to environmental stimuli [15]. In cancer therapy, nanozymes have been utilized to mediate chemodynamic therapy, photodynamic therapy, photothermal therapy, and radiotherapy, leveraging their ability to generate reactive oxygen species (ROS) and modulate the tumor microenvironment (TME) [16,17]. Moreover, nanozymes have shown promise in the development of biosensors, diagnostics, and components of drug delivery systems, highlighting their versatility in healthcare applications [13,18]. Recently, Cu single-atom nanozymes were reported to trigger cuproptosis and inhibit tumor growth and metastasis [19,20]. However, the current efficacy of single Cu-based nanozymes for inducing cuproptosis is inadequate since a high intracellular copper concentration is essential for inducing cuproptosis [21,22]. Therefore, a strategy involving the combination of Cu-based nanozymes with copper ionophores to improve the efficacy of cuproptosis induction is urgently needed.

**Fig. 1:**
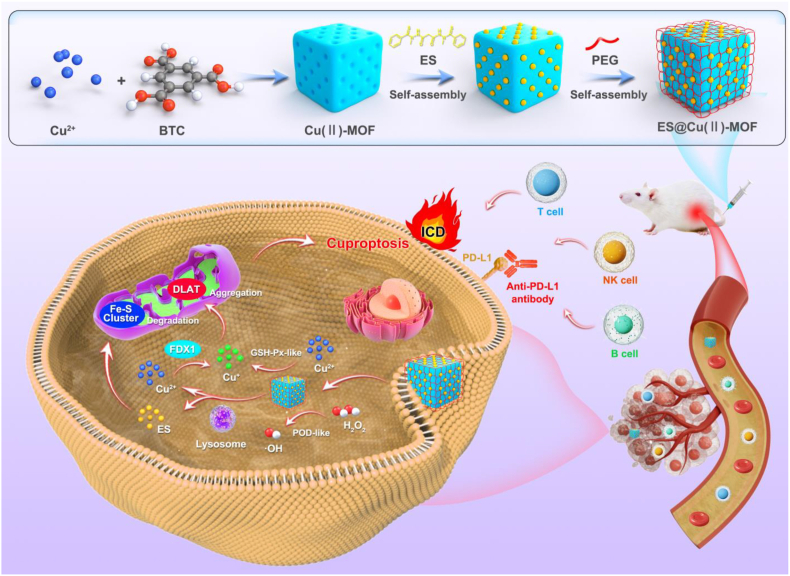
Schematic illustration of the synthesis of ES@Cu(Ⅱ)-MOF nanozymes and the mechanism of ES@Cu(Ⅱ)-MOF-induced cuproptosis for enhancing combined immunotherapy.

Metal‒organic frameworks (MOFs) are a class of crystalline materials known for their high porosity, large surface area, and tunable structure [23]. MOFs are synthesized through the self-assembly of metal ions or clusters with organic ligands, and the resulting materials have potential applications in various fields, such as drug delivery, gas catalysis, electrochemical sensing, and imaging [24]. One notable MOF, the copper-based MOF (Cu(Ⅱ)-MOF), has been extensively studied for its biomedical applications [25]. The incorporation of copper ions in Cu(Ⅱ)-MOFs endows them with unique activities [26,27] that are beneficial for inducing cuproptosis in cancer cells [28]. Despite this substantial progress, the detailed nanozyme action mechanisms, optimized induction efficiency, and biocompatibility of Cu(Ⅱ)-MOFs for cuproptosis induction have not been determined. In this study, we synthesized a Cu(Ⅱ)-MOF nanocomposite loaded with the copper ionophore ES and surface encapsulated with polyethylene glycol polymer (PEG) (denoted ES@Cu(Ⅱ)-MOF) to effectively activate cuproptosis. The obtained ES@Cu(Ⅱ)-MOF nanoparticles (NPs) exhibited outstanding enzyme-like activity. ES@Cu(Ⅱ)-MOF effectively converted H_2_O_2_ to hydroxyl radicals (•OH) via a highly efficient POD-like reaction. Moreover, ES@Cu(Ⅱ)-MOF NPs exhibited glutathione peroxidase (GSH-Px)-like enzymatic activity and could function as effective GSH consumers by converting Cu^2+^ to more toxic Cu^+^ ions. After internalization by mouse 4T1 breast cancer cells, Cu^+^ coupled with ES induced DLAT oligomerization and the downregulation of Fe‒S cluster proteins, such as ferredoxin 1 (FDX1) and dihydrolipoamide dehydrogenase (DLD) [3], ultimately leading to cuproptosis. In addition to the catalytic activity of ES@Cu(Ⅱ)-MOF, ES@Cu(Ⅱ)-MOF-induced cuproptosis led to significant inhibition of 4T1 cell growth *in vitro* and tumor growth *in vivo*. In addition, ES@Cu(Ⅱ)-MOF NPs effectively triggered immunogenic cell death (ICD), further activating the antitumor immune response. The combination of ES@Cu(Ⅱ)-MOF and programmed cell death-ligand 1 (PD-L1)-targeted therapy subsequently resulted in TME remodeling and increased T, B, and natural killer (NK) cell infiltration, thereby resulting in effective tumor ablation without obvious toxicity to normal tissues. Together, our results demonstrated the promising application of Cu-based nanozymes for cuproptosis-mediated antitumor therapy (Fig. 1).

## 2 Experimental section

### 2.1 Synthesis of Cu(Ⅱ)-MOF NPs

1.5 mL of 0.1 M Cu(NO_3_)_2_·3H_2_O aqueous solution and 1 mL of 0.1 M benzene-1,3,5-tricarboxylate (BTC) triethylammonium salt aqueous solution were added to 50 mL of a 1:1 (v/v) mixture of ethanol and ultrapure water [26,27]. The mixture was then subjected to vigorous stirring for 10 min at room temperature. The product was collected via centrifugation (12000×*g*, 10 min) and subsequently washed with anhydrous ethanol 3 times. The purified products were preserved in anhydrous ethanol at room temperature.

### 2.2 Synthesis of the ES@Cu(Ⅱ)-MOF NPs

ES (5 mg in 0.5 mL of DMSO) and Cu(Ⅱ)-MOF NPs (5 mg in 4.5 mL of ultrapure water) were mixed and stirred for 12 h. Subsequently, PEG_2000_ (5 mg) was incorporated into the mixture, and the mixture was stirred for an additional 12 h period. The mixture was then subjected to centrifugation (12000×*g*, 10 min), and the precipitate was washed with ultrapure water 3 times to remove impurities. The preparation of FTIC@Cu(Ⅱ)-MOF NPs and IR820@Cu(Ⅱ)-MOF NPs was carried out via similar methods.

### 2.3 Characterizations

The morphology of the nanozymes was observed via SEM (ZEISS Gemini SEM 300, Germany). The hydrodynamic size and zeta potential of the nanozymes were determined via a Zetasizer Nano Analyzer (Malvern, U.K.). XPS was carried out via an XPS instrument (Thermo Scientific K-Alpha, USA). XRD spectroscopy was carried out with an XRD spectrometer (Rigaku Ultima IV, Japan). UV–vis absorption spectra were measured by a UV–vis spectrometer (MAPADA, Shanghai). FTIR spectra were recorded on an FTIR spectrometer (Thermo Scientific Nicolet iS20, USA). N_2_ adsorption‒desorption isotherms were measured with a Micromeritics ASAP 2460 (USA).

### 2.4 Drug loading efficiency

The standard absorbance‒concentration calibration curve of ES was measured by a UV–Vis spectrophotometer at a wavelength of 390 nm. The encapsulation efficiency of ES was quantified by collecting the free ES remaining in the filtrate after centrifugation (12000×*g*, 10 min). The drug loading content (LC%) and entrapment efficiency (EE%) of the ES were calculated according to the following formulas:

LC % = M_Encapsulated ES mass_/M_Initial ES mass_ × 100%;

EE % = M_Encapsulated ES mass_/M _Drug-loaded nanocomplex mass_ × 100%.

### 2.5 pH-responsive degradation

ES@Cu(Ⅱ)-MOF NPs were added to PBS solutions (pH = 4.5, 5.5, and 7.5) under horizontal shaking at 100 rpm at room temperature. At the indicated time points, the solution was centrifuged at 12000×*g* for 10 min, and 100 μL of the supernatant was extracted. The released Cu and ES contents were quantified via ICP‒MS (Agilent 7800, USA) and HPLC (Agilent HPLC 1260, USA), respectively.

### 2.6 Measurement of POD-like activity

The concentration-dependent analysis of the POD-like activity of ES@Cu(Ⅱ)-MOF NPs was conducted by adding different concentrations of ES@Cu(Ⅱ)-MOF NPs to NaAc buffer. The mixture was subsequently incubated with 3 mM H_2_O_2_ and 1 mM TMB [29]. After incubation for 5 min, the absorbance at a wavelength of 652 nm was measured to assess the activity. ESR spectroscopy was used to further confirm the generation of •OH. After incubation with 3 mM 5,5-dimethyl-1-pyrroline-N-oxide (DMPO) or 1 mM H_2_O_2_ with ES@Cu(Ⅱ)-MOF NPs (10 μg/mL) for 10 min, ESR spectra were measured with an ESR spectrometer (Bruker EMXplus-6/1, Germany). The kinetic analysis of ES@Cu(Ⅱ)-MOF NPs (10 μg/mL) was performed using H_2_O_2_ as the substrate by adding 1 mM TMB and different amounts (0.1, 0.5, 1.0, 2.5, or 5.0 mM) of H_2_O_2_ solution. The absorbance of oxTMB was recorded by a UV–vis spectrophotometer at 652 nm (*ε* = 39000 M^−1^ cm^−1^) [22,30].

### 2.7 Measurement of GSH-Px-like activity

The GSH-Px-like activity of the ES@Cu(Ⅱ)-MOF nanozymes was assessed by using DTNB as the chromogenic substrate. The ES@Cu(Ⅱ)-MOF NPs were incubated with GSH (2 mM) and H_2_O_2_ (1.5 mM) in the dark. After incubation for the indicated durations, the reaction mixture was blended with DTNB (1 mM). After incubation for 1 min in the dark, the absorbance at a wavelength of 412 nm was measured to determine the enzymatic activity [31]. The kinetic analysis of ES@Cu(Ⅱ)-MOF NPs (10 μg/mL) was performed as catalysts in the presence of DTNB (1 mM), H_2_O_2_ (1.5 mM) and various concentrations of GSH (0.5, 1.0, 2.0, 4.0, or 6.0 mM). The absorbance was recorded with a UV–vis spectrophotometer (*ε* = 13600 M^−1^ cm^−1^ for TNB) [22,30].

### 2.8 Cell culture

Murine breast cancer cells (4T1 cells), murine macrophages (RAW264.7), human triple-negative breast cancer cell lines (MDA-MB-231 and MDA-MB-468) and murine brain-derived endothelial cells (BEND3) were cultured in Dulbecco's modified Eagle's medium (DMEM) or Roswell Park Memorial Institute (RPMI) 1640 medium, both of which contained 10 % fetal bovine serum (FBS) and 1 % penicillin/streptomycin. The cells were maintained in a cell incubator (Thermo Scientific, USA) at 37 °C with a 5 % CO_2_ atmosphere.

### 2.9 Cellular uptake

4T1 cells were seeded into glass-bottom cell culture dishes at a density of 1 × 10^5^ cells per dish and incubated overnight. The cells were subsequently treated with FITC@Cu(Ⅱ)-MOF NPs (1 μg/mL) for 8 h. After that, the cells were stained with Hoechst 33342 and LysoTracker for 10 min. The fluorescence was analyzed via CLSM (Leica, Germany) or a flow cytometer (Beckman Coulter, USA).

### 2.10 Intracellular Cu content detection

4T1 cells were seeded into 6-well plates at a density of 1 × 10^6^ cells per well and cultured for 12 h. The cells were then incubated with FITC@Cu(Ⅱ)-MOF NPs (1 μg/mL) for 8 h. After incubation, the cells were washed with PBS three times. The cells were subsequently lysed in a 2 % nitric acid solution at 65 °C for 1 h [3]. The obtained lysates were subsequently resuspended in ultrapure water, and the intracellular Cu content was quantified via an ICP‒MS instrument (Agilent 7800, USA).

### 2.11 Calcein-AM/PI staining

4T1 cells were seeded into glass-bottom cell culture dishes at a density of 1 × 10^5^ cells per dish and incubated overnight. The cells were subsequently treated with various medium containing PBS, 1 μM CuCl_2_, 50 nM ES, 1 μM CuCl_2_+50 nM ES, 0.4 μg/mL Cu(Ⅱ)-MOF NPs or 0.4 μg/mL ES@Cu(Ⅱ)-MOF NPs. After incubation for 24 h, the cells were stained with calcein-AM and PI for 10 min. The fluorescence images were acquired via CLSM (Leica, Germany).

### 2.12 GSH detection

4T1 cells were seeded in a 6-well plate at a density of 1 × 10^5^ cells per well and incubated overnight. Then, the cells were treated with fresh medium containing PBS, 1 μM CuCl_2_, 50 nM ES, 1 μM CuCl_2_+50 nM ES, 0.4 μg/mL Cu(Ⅱ)-MOF NPs or 0.4 μg/mL ES@Cu(Ⅱ)-MOF NPs. After incubation for 24 h, the intracellular GSH levels were analyzed with a GSH kit according to the manufacturer's protocols.

### 2.13 Cell apoptosis assay

4T1 cells were seeded in 12-well plates at a density of 5 × 10^5^ cells per well and incubated overnight. The culture medium was subsequently replaced with fresh medium containing PBS, 1 μM CuCl_2_, 50 nM ES, 1 μM CuCl_2_+50 nM ES, 0.4 μg/mL Cu(Ⅱ)-MOF NPs or 0.4 μg/mL ES@Cu(Ⅱ)-MOF NPs. The cells were incubated for 24 h and then stained with Annexin V-FITC and PI apoptosis detection kits according to the manufacturer's instructions. Flow cytometric analysis was performed via a flow cytometer (Beckman Coulter, USA).

### 2.14 Colony formation assay

4T1 cells were seeded in 12-well plates at a density of 600 cells per well and cultured for 4 days. The cell colonies were subsequently treated with fresh medium containing PBS, 1 μM CuCl_2_, 50 nM ES, 1 μM CuCl_2_+50 nM ES, 0.4 μg/mL Cu(Ⅱ)-MOF NPs or 0.4 μg/mL ES@Cu(Ⅱ)-MOF NPs. After incubation for 2 days, the colonies were fixed with a 4 % paraformaldehyde solution for 10 min, followed by crystal violet staining for 15 min.

### 2.15 LDH and ATP assays

4T1 cells were seeded into 96-well plates at a density of 2 × 10^4^ cells per well and incubated overnight. The cells were subsequently treated with PBS, 1 μM CuCl_2_, 50 nM ES, 1 μM CuCl_2_+50 nM ES, 0.4 μg/mL Cu(Ⅱ)-MOF NPs or 0.4 μg/mL ES@Cu(Ⅱ)-MOF NPs. After incubation for 24 h, the extracellular LDH and intracellular ATP levels were measured via the LDH and ATP Assay Kit according to the manufacturer's protocol.

### 2.16 Western blot

The treated cells were lysed with RIPA buffer, and the proteins were extracted via centrifugation (12000×*g*, 15 min). The protein concentration was quantified with a BCA protein assay kit. Protein samples (40 μg) were separated via 12 % sodium dodecyl sulfate‒polyacrylamide gel electrophoresis (SDS‒PAGE) and then transferred to polyvinylidene fluoride (PVDF) membranes via gel electrophoretic equipment (Bio-Rad, USA). The PVDF membranes were blocked with a 5 % nonfat milk solution for 1 h, followed by an overnight incubation at 4 °C with anti-FDX1 or anti-DLD antibodies at a dilution of 1:1000. The membranes were subsequently rinsed with TBST three times and further incubated with an HRP-conjugated secondary antibody for 2 h at room temperature. After incubation with enhanced chemiluminescence (ECL) reagent (Millipore), western blot images were captured via a Gel Doc XR^+^ Gel Documentation System (Bio-Rad, USA). β-Actin was used as a protein loading control.

### 2.17 CCK-8 assay

Cell viability was measured via a CCK-8 kit according to the manufacturer's protocols. Breast cancer cells (4T1, MDA-MB-231, MDA-MB-468 or BEND3) were seeded in 96-well plates at a density of 2 × 10^4^ cells per well and cultured overnight. The cells were then treated with various NPs concentrations for 24 h. CCK-8 reagent was added to each well, and the OD values at 450 nm were measured via a microplate spectrophotometer (Tecan, Switzerland).

### 2.18 Intracellular ROS detection

4T1 cells were cultured in glass-bottom cell culture dishes at a density of 1 × 10^5^ cells per dish and cultured overnight. After the indicated treatments for 24 h, the cells were incubated with DCFH-DA and Dil for 15 min [32]. Subsequently, fluorescence images were obtained via CLSM (Leica, Germany).

### 2.19 Immunofluorescence

After the indicated treatments, the cells were fixed with a 4 % paraformaldehyde solution for 10 min and subsequently blocked with 5 % BSA containing 0.25 % Triton X-100 for 20 min. The cells were incubated overnight with anti-HMGB-1 (dilution 1:200) or anti-CRT antibodies (dilution 1:200) at 4 °C. Then, the cells were stained with Alexa Fluor™ 488-labeled secondary antibody (dilution 1:300) for 1 h, and the nuclei were stained with DAPI for 10 min at room temperature. Images were obtained via CLSM (Leica, Germany).

For immunofluorescence of DLAT foci, the cells subjected to the indicated treatments were stained with MitoTracker at 37 °C for 20 min [3]. Afterward, the cells were fixed with 4 % paraformaldehyde for 10 min and subsequently blocked with 5 % BSA solution containing 0.25 % Triton X-100 for 20 min. The cells were then incubated with the anti-DLAT antibody (dilution 1:200) overnight at 4 °C, followed by incubation with the Alexa Fluor™ 488-labeled secondary antibody at room temperature for 1 h. After washing three times with TBST, the nuclei were finally stained with DAPI for 10 min at room temperature. Images were captured via CLSM (Leica, Germany), and the fluorescence intensity was quantified via ImageJ software (NIH, Bethesda, MD).

### 2.20 Transwell migration assay

4T1 cells were seeded in 6-well plates at a density of 1 × 10^6^ cells per well and incubated overnight. The cells were incubated with fresh medium containing PBS, 1 μM CuCl_2_, 50 nM ES, 1 μM CuCl_2_+50 nM ES, 0.4 μg/mL Cu(Ⅱ)-MOF NPs or 0.4 μg/mL ES@Cu(Ⅱ)-MOF NPs. After incubation for 24 h, the medium was collected and centrifuged at 12000×*g* for 20 min at 4 °C. The conditioned medium was added to the lower chamber, and RAW264.7 macrophages (1 × 10^5^) were added to the upper chamber [33,34]. After 24 h of incubation, the migrated RAW264.7 cells were stained with crystal violet and observed via an inverted microscope.

### 2.21 *In vivo* antitumor study

Female BALB/c mice (4 weeks old) were obtained from Vital River Laboratory Animal Technology Co., Ltd. Mice were housed in specific-pathogen-free (SPF) rodent facilities. The animal study was approved by the Institutional Animal Care and Use Committee of Wenzhou Institute, University of Chinese Academy of Sciences (Approval number: WIUCAS22110102). To establish a 4T1 tumor-bearing model, a 4T1 cell suspension (1 × 10^6^) was inoculated into the right flanks of the mice. On day 6, the mice were randomly divided into six distinct groups. On days 6, 9, 12 and 15, the mice were treated with PBS, Cu(Ⅱ)-MOF NPs (100 μg/mouse), ES (93 μg/mouse), ES@Cu(Ⅱ)-MOF NPs (100 μg/mouse), anti-PD-L1 antibody (100 μg/mouse), or ES@Cu(Ⅱ)-MOF NPs (100 μg/mouse) + Anti-PD-L1 (100 μg/mouse) (n = 5). The tumor volume and body weight were measured every three days, and the tumor volume was calculated via the following formula: tumor volume (mm^3^) = (length × width^2^)/2. After treatment, blood samples and tissues from the heart, liver, spleen, lung, kidney, and tumors were collected for further analysis. For the survival study, the mice were humanely sacrificed once their tumors reached or exceeded a volume of 2000 mm^3^.

### 2.22 *In vivo* pharmacokinetics profiles

4T1 tumor-bearing mice were intravenously injected with IR820 or IR820@Cu(Ⅱ)-MOF NPs (2 μg/mouse). *In vivo* fluorescence imaging was conducted at the indicated time points via the IVIS Lumina III system (Perkin Elmer, USA). At 48 h postinjection, the mice were euthanized, and both the tumor tissues and main organs were extracted for subsequent biodistribution imaging assessments.

### 2.23 Flow cytometry analysis

The excised tumors were incubated in RPMI 1640 medium containing 1 mg/mL collagenase IV, 1 mg/mL hyaluronidase, 0.1 mg/mL DNase I, and 2 % FBS at 37 °C for 20 min. The tumors were mechanically dissociated via a gentleMACS™ Dissociator (Miltenyi Biotech) and then subjected to Percoll gradient centrifugation to isolate a single-cell suspension of lymphocytes. The cells were subsequently stained with a series of antibodies: TruStain FcX antibody, LIVE/DEAD fixable reagent, anti-CD45 antibody, anti-CD3 antibody, anti-CD4 antibody, anti-CD8 antibody, anti-CD49b antibody, anti-B220 antibody, and anti-FOXP3 antibody, according to the manufacturer's protocol. The flow cytometric analyses were performed on a flow cytometer (Beckman Coulter, USA), and the data were analyzed and quantified via FlowJo software (version 10.9, Ashland, USA).

### 2.24 Histopathological analysis

The excised tumors and organs were fixed in 4 % paraformaldehyde solution, followed by embedding in paraffin. The samples were then sectioned and subjected to H&E staining. For IHC staining, the sections were treated with retrieval buffer (pH = 6.0) and then blocked with 3 % H_2_O_2_ solution for 10 min at room temperature to block endogenous peroxidase activity. After permeabilization with 0.25 % Triton X-100 solution, the slides were incubated with 5 % goat serum for 30 min at room temperature to reduce nonspecific binding. The sections were then incubated with anti-FDX1 (dilution 1:100), anti-CD8 (dilution 1:100), or anti-Ki67 (dilution 1:200) antibodies at 4 °C overnight. After that, the slides were treated with a secondary antibody (Dako REAL™ EnVision™ detection system) for 1 h at room temperature. After being washed with TBST, the slides were developed with 3,3′-diaminobenzidine (DAB) to visualize the immunoreactive sites. The slides were then counterstained with hematoxylin, dehydrated through a series of alcohol concentrations, and then examined via a microscope for imaging.

### 2.25 ELISA

The ocular venous blood from the mice was allowed to coagulate naturally at 4 °C for 2 h and then centrifuged at 2000×*g* for 20 min at 4 °C to separate the serum from the blood samples. The levels of blood biochemistry and cytokines were subsequently determined via ELISA kits according to the manufacturer's methods.

### 2.26 Statistical analysis

All the statistical analyses were conducted via GraphPad Prism 10 (GraphPad Software Inc., USA). The data are presented as the means ± SDs. For comparisons between two groups, the data were evaluated via two-tailed Student's t tests. Survival data were examined via the Kaplan‒Meier method, and the significance of the differences was tested via the log-rank test. A difference was regarded as significant when the p value was less than or equal to 0.05. ∗p < 0.05, ∗∗p < 0.01, ∗∗∗p < 0.001, ns, not significant.

## 3 Results and discussion

### 3.1 Preparation of ES@Cu(Ⅱ)-MOF NPs

**Fig. 2:**
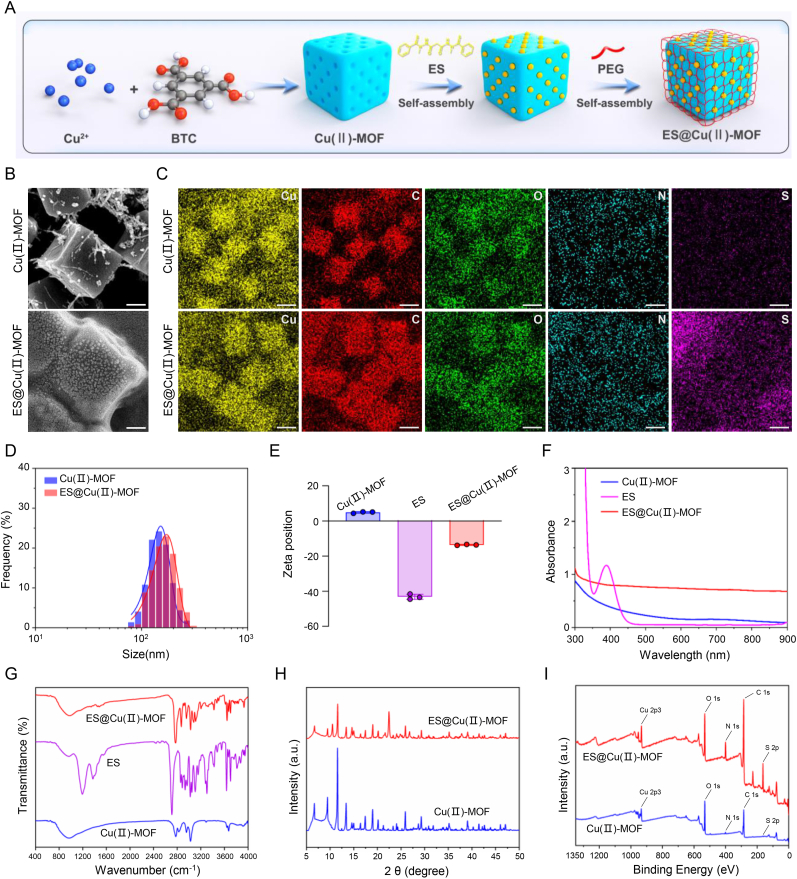
Characteristics of the ES@Cu(Ⅱ)-MOF NPs. A) Schematic illustration of the synthesis of the ES@Cu(Ⅱ)-MOF NPs. B) SEM images of the Cu(Ⅱ)-MOF NPs and ES@Cu(Ⅱ)-MOF NPs. Scale bar = 50 nm. C) Elemental mapping (Cu, C, N, O, and S) of the Cu(Ⅱ)-MOF NPs and ES@Cu(Ⅱ)-MOF NPs. Scale bar = 100 nm. D) Hydrodynamic sizes of the Cu(Ⅱ)-MOF NPs (average size = 152.7 nm, polydispersity index = 0.13) and ES@Cu(Ⅱ)-MOF NPs (average size = 171.9 nm, polydispersity index = 0.18). E) Zeta potentials of the Cu(Ⅱ)-MOF NPs, ES, and ES@Cu(Ⅱ)-MOF NPs (n = 3). F) UV–vis absorption spectra of the Cu(Ⅱ)-MOF NPs, ES, and ES@Cu(Ⅱ)-MOF NPs. G) FTIR spectra of the Cu(Ⅱ)-MOF NPs, ES, and ES@Cu(Ⅱ)-MOF NPs. H) XRD patterns of the Cu(Ⅱ)-MOF NPs and ES@Cu(Ⅱ)-MOF NPs. I) XPS analysis of the Cu(Ⅱ)-MOF NPs and ES@Cu(Ⅱ)-MOF NPs. The chemical formula of ES is C_19_H_20_N_4_O_2_S_2_. The data are presented as the means ± SDs.

In brief, Cu(Ⅱ)-MOF NPs were synthesized via self-assembly of Cu^2+^ and benzene-1,3,5-tricarboxylate triethylammonium salt in a mixture of ethanol and ultrapure water [26,27]. The N_2_ adsorption/desorption results revealed that the Brunauer–Emmett–Teller (BET) specific surface area of the Cu(Ⅱ)-MOF NPs was ∼1599 m^2^/g and that the pore size was ∼2 nm (Fig. S1), which is ideal for the diffusion of ES through the framework. Then, the Cu(Ⅱ)-MOF NPs were modified with the copper ionophore ES through the chelation of Cu^2+^-ES [35], and PEG was used to encapsulate the ES-Cu(Ⅱ)-MOF NPs to increase their physiological compatibility (Fig. 2A; Fig. S2). Scanning electron microscopy (SEM) images revealed that the synthesized ES@Cu(Ⅱ)-MOF NPs had a monodispersed cubic morphology and a membrane structure (Fig. 2B–D). In addition, the successful construction of the ES@Cu(Ⅱ)-MOF NPs was further confirmed by elemental mapping and energy-dispersive spectroscopy analysis (Fig. 2C; Fig. S3). After ES loading, the zeta potential of the Cu(Ⅱ)-MOF NPs decreased from ∼4.8 mV to ∼ −13.6 mV, verifying the successful binding of ES (Fig. 2E). The successful generation of ES@Cu(Ⅱ)-MOF NPs was further verified by both ultraviolet–visible (UV–vis) spectrophotometry and Fourier transform infrared (FTIR) spectroscopy analysis (Fig. 2F and G). The drug loading content (LC%) and entrapment efficiency (EE%), which were calculated via UV–vis spectrophotometry at the characteristic peak (390 nm) of ES, were ∼93 % and ∼48 %, respectively. The inductively coupled plasma‒mass spectrometry (ICP‒MS) results revealed that the Cu content of ES@Cu(Ⅱ)-MOF NPs was ∼16 %, indicating the preserved catalytic capability of ES@Cu(Ⅱ)-MOF NPs. Furthermore, X-ray diffraction (XRD) analysis of the synthesized ES@Cu(II)-MOF NPs revealed a pattern identical to that of the pristine Cu(II)-MOF NPs. (Fig. 2H). X-ray photoelectron spectroscopy (XPS) results suggested that Cu, C, N, and O were present in the Cu(Ⅱ)-MOF NPs, whereas the binding energy peak was shifted in the N 1s, O 1s, and S 2p spectra of the ES@Cu(Ⅱ)-MOF NPs. Furthermore, the ES@Cu(Ⅱ)-MOF NPs contained a large amount of S, which provided direct evidence for the successful loading of ES onto the Cu(Ⅱ)-MOF NPs (Fig. 2I; Fig. S4).

### 3.2 pH-responsive release profile of ES@Cu(Ⅱ)-MOF NPs

**Fig. 3:**
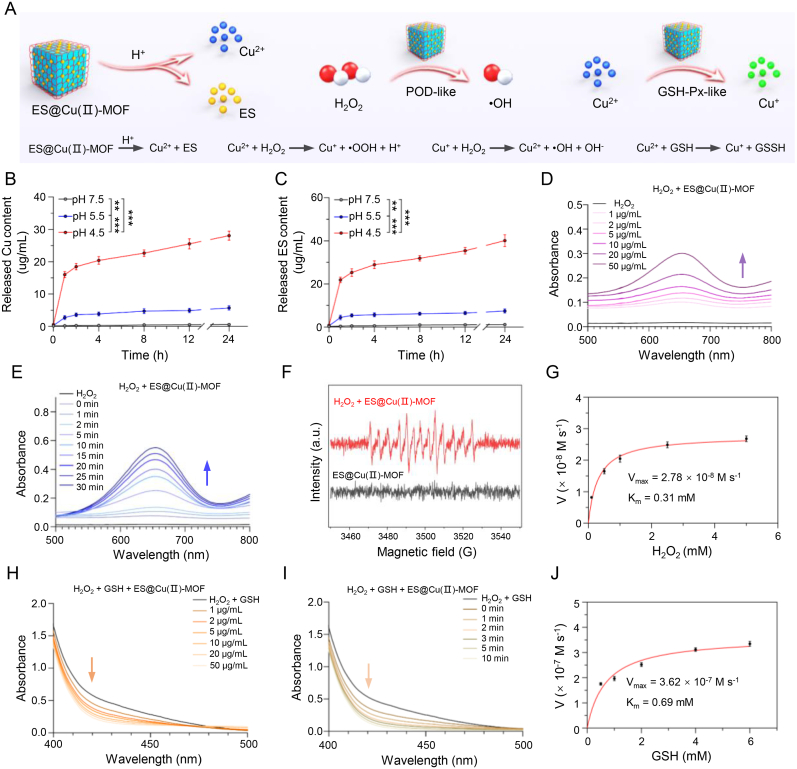
The nanozyme activity of the ES@Cu(Ⅱ)-MOF NPs. A) Schematic illustration of the nanozyme activity of the ES@Cu(Ⅱ)-MOF NPs. B, C) Cumulative release of Cu and ES from the ES@Cu(Ⅱ)-MOF NPs in PBS at pH values of 4.5, 5.5, and 7.5, respectively (n = 3). D, E) Analyses of the POD-like activities of ES@Cu(Ⅱ)-MOF NPs treated with various concentrations of ES@Cu(Ⅱ)-MOF NPs and reaction times using TMB as a substrate. F) •OH generation detected by ESR spectroscopy. DMPO was used as a spin trap for •OH. G) Michaelis‒Menten kinetic analysis of ES@Cu(Ⅱ)-MOF NPs with H_2_O_2_ as a substrate. H, I) Analyses of the GSH-Px-like activities of ES@Cu(Ⅱ)-MOF NPs treated with various concentrations of ES@Cu(Ⅱ)-MOF NPs and reaction times using DTNB as a substrate. J) Michaelis‒Menten kinetic analysis for ES@Cu(Ⅱ)-MOF NPs with GSH as a substrate. The data are presented as the means ± SDs; ∗∗p < 0.01; ∗∗∗p < 0.001.

Given that Cu^2+^ and ES are essential for the efficient induction of cuproptosis [3], we first measured the concentration of Cu^2+^ released by the ES@Cu(Ⅱ)-MOF nanocomposite. ES@Cu(Ⅱ)-MOF NPs released ∼0.3 % of the Cu^2+^ after 24 h of incubation in pH 7.5 phosphate-buffered saline (PBS), indicating the relative stability of the ES@Cu(Ⅱ)-MOF NPs in physiological environments. In contrast, the ES@Cu(Ⅱ)-MOF NPs exhibited obvious pH-responsive biodegradation behavior in PBS at pH 5.5 and pH 4.5, having released ∼3.6 % and ∼17.5 % of the Cu^2+^, respectively, at 24 h (Fig. 3A and B). Moreover, the release of ES from the ES@Cu(Ⅱ)-MOF NPs under acidic conditions was demonstrated via high-performance liquid chromatography (HPLC). These results indicated that the ES released from the ES@Cu(Ⅱ)-MOF NPs occurred in a pH-sensitive manner (Fig. 3C). Together, the above results suggested that the ES@Cu(Ⅱ)-MOF NPs had favorable biodegradability in the acidic TME.

### 3.3 Nanozyme activities of ES@Cu(Ⅱ)-MOF NPs

The ES@Cu(Ⅱ)-MOF nanocomposite integrated the POD-like and GSH-Px-like activities of the Cu(Ⅱ)-MOF NPs [26,27], and the catalytic activities of the ES@Cu(Ⅱ)-MOF NPs were subsequently assessed. To show the POD-like activity of the ES@Cu(Ⅱ)-MOF nanozymes, we used 3,3,5,5-tetramethylbenzidine (TMB) as a probe to quantitatively analyze the generation of •OH. In this assay, colorless TMB was oxidized by •OH via a colorimetric reaction to generate a blue product (oxTMB) with a characteristic absorbance at 652 nm [29]. Compared with those of H_2_O_2_ alone, the absorbance peaks of oxTMB increased with increasing ES@Cu(Ⅱ)-MOF NPs concentration and reaction time, indicating that ES@Cu(Ⅱ)-MOF could catalyze H_2_O_2_ to generate a large amount of •OH (Fig. 3A; 3D and 3E). In addition, the POD-like catalytic activity of the ES@Cu(Ⅱ)-MOF nanozymes was pH dependent, further indicating their excellent POD-like activity (Fig. S5). Furthermore, an electron spin resonance (ESR) assay was used to further verify the production of •OH (Fig. 3F). The conversion of H_2_O_2_ by ES@Cu(Ⅱ)-MOF NPs followed the Michaelis–Menten process with Km and Vmax values of 0.31 mM and 2.78 × 10^−8^ M s^−1^, respectively (Fig. 3G).

GSH, an endogenous antioxidant, can inhibit ROS production and cuproptosis [3,36]. To demonstrate the GSH-Px-like capability of the ES@Cu(Ⅱ)-MOF NPs, 5,5′-dithiobis-(2-nitrobenzoic acid) (DTNB) was used as a specific GSH indicator to examine whether the ES@Cu(Ⅱ)-MOF NPs had GSH-Px-like activity. The colorless compound DTNB can react with the residual thiol of GSH to generate a yellow substance (2-nitro-5-thiobenzoic acid anion, TNB) with a characteristic peak at 412 nm [31]. The adsorption peak of TNB decreased with increasing ES@Cu(Ⅱ)-MOF NPs concentration and reaction time, indicating the excellent GSH depletion capability of the ES@Cu(Ⅱ)-MOF NPs (Fig. 3A; 3H and 3I; Fig. S5). The Michaelis–Menten equation revealed that the ES@Cu(Ⅱ)-MOF NPs had comparable Km (0.69 mM) and Vmax (3.62 × 10^−7^ M s^−1^) values (Fig. 3J). Together, the above results verified that the biodegradable ES@Cu(Ⅱ)-MOF NPs had POD-like and GSH-Px-like activities, which could augment the ability to induce cuproptosis by increasing ROS production and GSH consumption.

### 3.4 Intracellular uptake ability

**Fig. 4:**
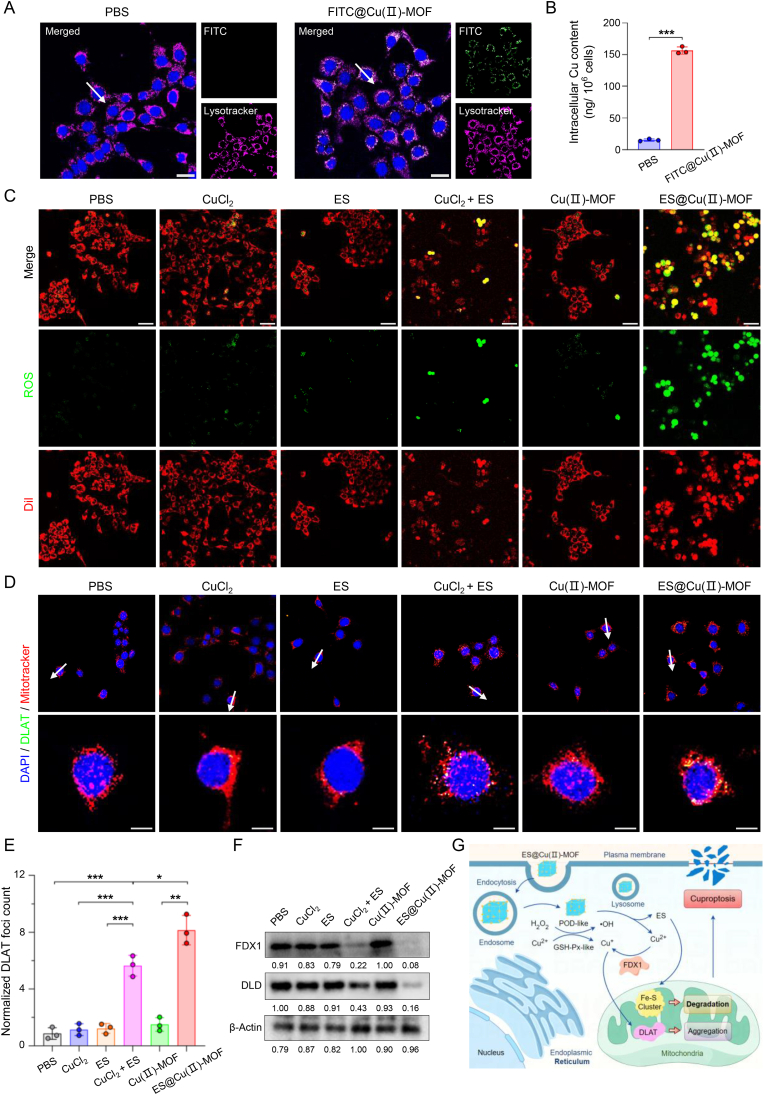
Effects of ES@Cu(Ⅱ)-MOF NPs on cuproptosis induction. A) CLSM imaging analysis of the cellular uptake of FITC@Cu(Ⅱ)-MOF NPs (green) in 4T1 cells after 8 h of incubation. The cell nuclei were stained with Hoechst 33342 (blue), and the lysosomes were stained with LysoTracker (pink). Scale bar: 25 μm. The fluorescence intensities of the FITC@Cu(Ⅱ)-MOF (green) and lysosome (pink) signals across the regions indicated with white arrows were analyzed and shown in line plots (Fig. S7). B) ICP‒MS analysis of the intracellular Cu content in 4T1 cells after 8 h of incubation (n = 3). C) CLSM images of the intracellular ROS level (green) in 4T1 cells following the indicated treatments. The cells were stained with Dil (red). Scale bar: 50 μm. D) CLSM images of DLAT foci (green) in 4T1 cells after the indicated treatment. The cell nuclei were stained with DAPI (blue), and the mitochondria were stained with MitoTracker (red). Scale bars: 10 μm. The fluorescence intensities of DLAT foci (green) and mitochondrial (red) signals across the regions indicated with white arrows were analyzed and shown in line plots (Fig. S10). E) Quantification of DLAT foci in 4T1 cells after the indicated treatment (n = 3). F) Western blot analysis of the expression of Fe‒S cluster proteins (FDX1 and DLD) in 4T1 cells after different treatments. The intensity of each protein band was quantified via NIH ImageJ software. G) Schematic illustration of the mechanism of ES@Cu(Ⅱ)-MOF nanozyme-induced cuproptosis. 4T1 cells treated with CuCl_2_ + ES were used as the positive control [3]. The data are presented as the means ± SDs; ∗p < 0.05; ∗∗p < 0.01; ∗∗∗p < 0.001.

To determine the intracellular uptake of the ES@Cu(Ⅱ)-MOF NPs, fluorescein isothiocyanate (FITC)-loaded Cu(Ⅱ)-MOF (FITC@Cu(Ⅱ)-MOF) NPs were used to monitor their cellular uptake in 4T1 cells. Flow cytometric analysis revealed that the fluorescence intensity in 4T1 cells treated with FITC@Cu(Ⅱ)-MOF NPs was markedly greater than that in cells treated with PBS for 8 h (Fig. S6). Furthermore, confocal laser scanning microscopy (CLSM) revealed that the intracellular distribution of the FITC fluorescence signal overlapped well with that of lysosomes in 4T1 cells (Fig. 4A; Fig. S7). As the intracellular copper concentration is an essential factor influencing cuproptosis induction [3,36], the accumulation of copper in 4T1 cells was quantified via ICP‒MS analysis. As shown in Fig. 4B, the intracellular content of copper (∼156.4 ng/10^6^ cells) in 4T1 cells treated with FITC@Cu(Ⅱ)-MOF NPs was greater than that in the control group (∼15.2 ng/10^6^ cells) after 8 h of incubation, indicating that ES@Cu(Ⅱ)-MOF NPs can be rapidly internalized by 4T1 cells via the endolysosomal pathway.

### 3.5 ES@Cu(Ⅱ)-MOF NPs triggered cuproptosis

Encouraged by the superior nanozyme activity of the ES@Cu(Ⅱ)-MOF NPs (Fig. 3), we next sought to determine the underlying mechanism of ES@Cu(Ⅱ)-MOF-induced cuproptosis. The intracellular level of ROS after treatment with the ES@Cu(Ⅱ)-MOF NPs was visualized by using 2,7-dichlorofluorescein diacetate (DCFH-DA), which can be oxidized by ROS to generate bright green fluorescence within tumor cells [32]. As expected, the CLSM images revealed that the fluorescence signal was strongest after ES@Cu(Ⅱ)-MOF treatment in 4T1 cells (Fig. 4C; Fig. S8). GSH, an endogenous copper chelator, sequesters intracellular Cu^2+^ to inhibit cuproptosis [3,36]. Owing to the GSH-Px-like activity of ES@Cu(Ⅱ)-MOF NPs (Fig. 3), the intracellular GSH level obviously decreased after ES@Cu(Ⅱ)-MOF NPs treatment (Fig. S9), further verifying the remarkable catalytic activity of the ES@Cu(Ⅱ)-MOF NPs in the intracellular environment. On the other hand, Cu^2+^ can be converted to more toxic Cu^+^ ions via FDX1, and DLAT can bind to Cu^+^ to induce DLAT aggregation and subsequently trigger cuproptosis [3]. The DLAT foci were subsequently evaluated via CLSM imaging. 4T1 cells treated with CuCl_2_+ES (positive control [3]) exhibited noticeable DLAT aggregation, while DLAT oligomerization was more obvious in the mitochondria of 4T1 cells after ES@Cu(Ⅱ)-MOF treatment (Fig. 4D and E; Fig. S10). Moreover, the expression of FDX1 and DLD, which are hallmarks of Fe‒S cluster proteins during cuproptosis [3], was significantly reduced in 4T1 cells after ES@Cu(Ⅱ)-MOF treatment (Fig. 4F). Together, these results showed that these ES@Cu(Ⅱ)-MOF NPs with excellent nanozyme activity effectively promoted the activation of cuproptosis (Fig. 4G).

### 3.6 ES@Cu(Ⅱ)-MOF-induced cuproptosis mediated cell death

**Fig. 5:**
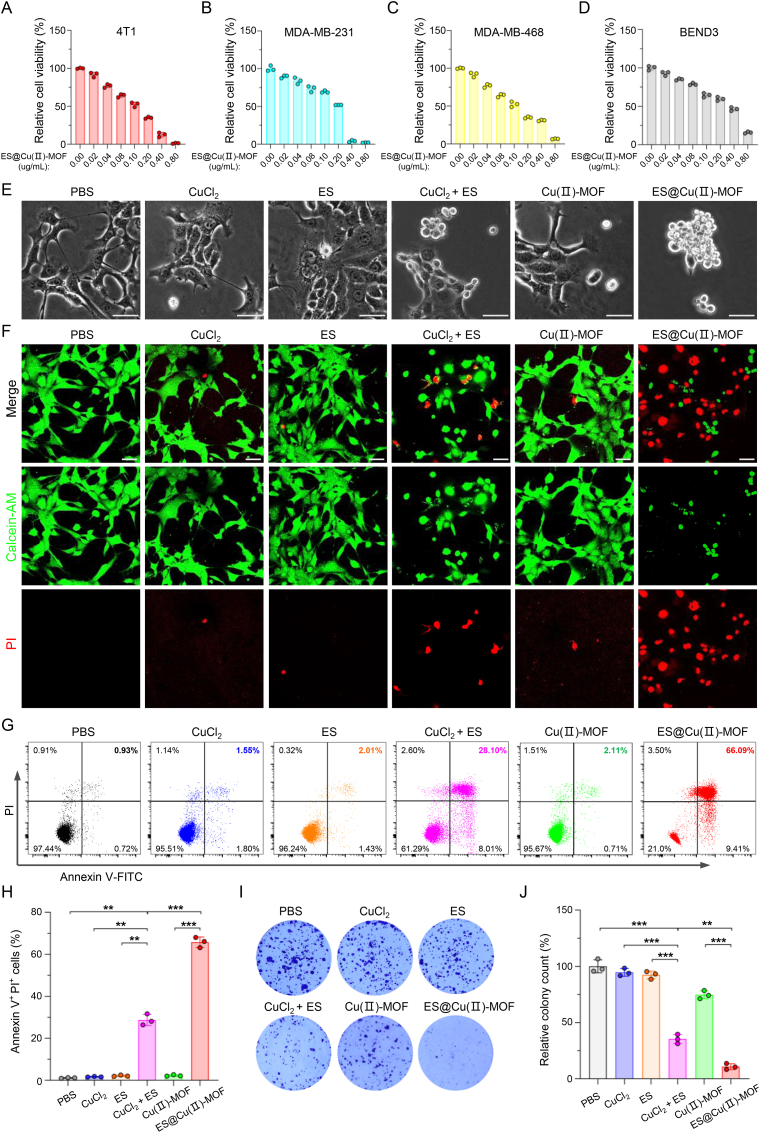
The antitumor effect of ES@Cu(Ⅱ)-MOF-induced cuproptosis *in vitro*. A-D) Relative viabilities of 4T1, MDA-MB-231, MDA-MB-468, and BEND3 cells following 24 h of incubation with a gradient concentration of ES@Cu(Ⅱ)-MOF NPs (n = 3). E) Morphological injury of 4T1 cells after different treatments. Scale bar: 50 μm. F) CLSM images of 4T1 cells stained with calcein-AM (green, live cells) and PI (red, dead cells) after different treatments. Scale bar: 50 μm. G) Flow cytometric analysis of the degree of necrosis in 4T1 cells following various treatments. H) Quantification of Annexin V-FITC^+^/PI^+^ 4T1 cells after the indicated treatment (n = 3). I) Colony formation ability of 4T1 cells after different treatments. J) Quantification of the number of colonies formed by 4T1 cells after the indicated treatment (n = 3). 4T1 cells treated with CuCl_2_ + ES were used as the positive control [3]. The data are presented as the means ± SDs; ∗∗p < 0.01; ∗∗∗p < 0.001.

Next, we analyzed the antitumor ability of ES@Cu(Ⅱ)-MOF NPs through a well-known cell counting kit-8 (CCK-8) assay. As expected, the viability of 4T1 cells obviously decreased following treatment with 100 μM CuCl_2_ or 100 μg/mL Cu(Ⅱ)-MOF alone (Fig. S11), further confirming that only a high level of Cu^2+^ triggers marked cuproptosis [3]. When the concentration of ES@Cu(Ⅱ)-MOF NPs was 0.40 μg/mL, the growth inhibition rate in the ES@Cu(Ⅱ)-MOF group exceeded 85 % (Fig. 5A). Consistent with this finding, ES@Cu(Ⅱ)-MOF NPs displayed a similar cytotoxic effect on human triple-negative breast cancer cell lines (MDA-MB-468 and MDA-MB-231), indicating that ES@Cu(Ⅱ)-MOF NPs have a broad-spectrum antitumor effect (Fig. 5B and C). However, approximately half of the murine brain-derived endothelial cells (BEND3) survived after 24 h of incubation at a dose of 0.40 μg/mL ES@Cu(Ⅱ)-MOF NPs, indicating the excellent biocompatibility of ES@Cu(Ⅱ)-MOF NPs (Fig. 5D). Additionally, the noticeable morphological changes further confirmed the cytotoxicity of the ES@Cu(Ⅱ)-MOF NPs (Fig. 5E). The antitumor effect of ES@Cu(Ⅱ)-MOF was subsequently verified via calcein-AM and propidium iodide (PI) staining. Cu(Ⅱ)-MOF NPs showed little cytotoxicity in 4T1 cells, indicating their good biocompatibility. In contrast, treatment with ES@Cu(Ⅱ)-MOF caused the death of numerous 4T1 cells (Fig. 5F; Fig. S12). Moreover, flow cytometric analysis revealed a significant presence of necrotic cells within the group treated with ES@Cu(II)-MOF NPs, further revealing the superior antitumor efficacy of ES@Cu(II)-MOF NPs (Fig. 5G and H). To verify the inhibitory effect of ES@Cu(Ⅱ)-MOF-induced cuproptosis on tumor growth, we also carried out a colony formation assay. As expected, ES@Cu(Ⅱ)-MOF obviously inhibited the formation of 4T1 tumor cell colonies (Fig. 5I and J). Together, these results showed that ES@Cu(Ⅱ)-MOF-triggered cuproptosis prominently contributed to the antitumor effect.

### 3.7 ES@Cu(Ⅱ)-MOF-triggered ICD

**Fig. 6:**
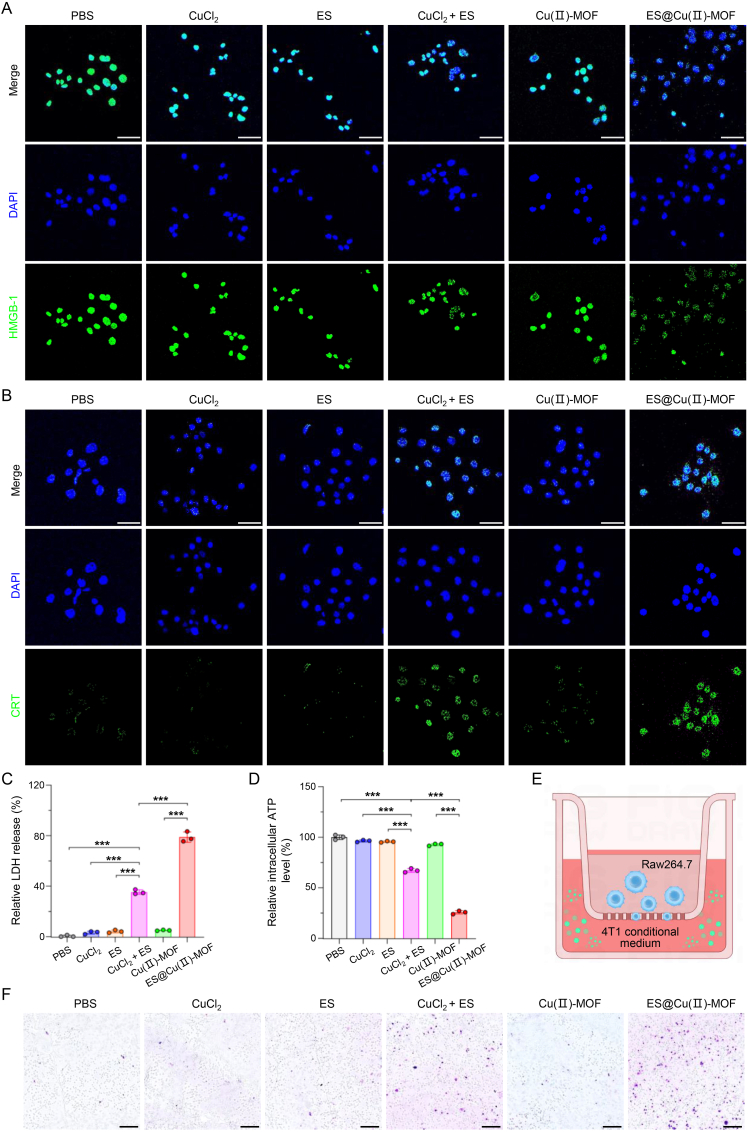
ICD triggered by ES@Cu(Ⅱ)-MOF NPs *in vitro*. A) CLSM images of the released HMGB-1 (green) in 4T1 cells after the indicated treatment. The cell nuclei were stained with DAPI (blue). Scale bars: 50 μm. B) CLSM images of exposed CRT (green) after the indicated treatment. The cell nuclei were stained with DAPI (blue). Scale bars: 50 μm. C) Released LDH levels in 4T1 cells after the indicated treatments (n = 3). D) Intracellular ATP levels in 4T1 cells after the indicated treatments (n = 3). E) Schematic illustration of the transwell experiment. The conditioned medium following the indicated treatments was added to the lower chamber, and RAW264.7 macrophages were cultured in the upper chamber. F) Chemotaxis of RAW264.7 macrophages stimulated with the indicated conditioned medium. Scale bars: 200 μm. 4T1 cells treated with CuCl_2_ + ES were used as the positive control [3]. The data are presented as the means ± SDs; ∗∗∗p < 0.001.

To evaluate the ES@Cu(Ⅱ)-MOF-mediated antitumor immune response *in vitro*, damage-associated molecular pattern (DAMP) molecules, including high mobility group box-1 (HMGB-1), calreticulin (CRT), adenosine triphosphate (ATP), and lactate dehydrogenase (LDH) [37,38], were subsequently analyzed. The release of HMGB-1 and exposure to CRT after different treatments were evaluated via CLSM imaging. CuCl_2_+ES (positive control [3]) induced only slight HMGB-1 release and CRT exposure. Notably, ES@Cu(Ⅱ)-MOF treatment led to the most significant increases in HMGB-1 release and CRT exposure among all the groups due to the efficient induction of cuproptosis by ES@Cu(Ⅱ)-MOF NPs in 4T1 cells (Fig. 6A and B; Figs. S13 and S14). Consistent with this finding, the release of LDH and ATP in the extracellular environment after ES@Cu(Ⅱ)-MOF treatment was obviously greater than that in the other groups, indicating that ES@Cu(Ⅱ)-MOF-mediated cuproptosis effectively enhanced the release of DAMPs into the TME (Fig. 6C and D). DAMPs can function as “find me” signals or “eat me” signals to increase the recruitment of antigen-presenting cells to tumor cells [33,34]. The immune response was further evaluated via a transwell assay (Fig. 6E). When the murine macrophage cell line RAW264.7 was stimulated with ES@Cu(Ⅱ)-MOF-treated 4T1 cell-conditioned medium, an abundance of migrated RAW264.7 cells was observed, which demonstrated that ES@Cu(Ⅱ)-MOF dramatically increased the immunogenicity of 4T1 cells (Fig. 6F; Fig. S15). Overall, the above results revealed that ES@Cu(Ⅱ)-MOF-triggered cuproptosis augmented the antitumor immune response.

### 3.8 Biodistribution analysis *in vivo*

**Fig. 7:**
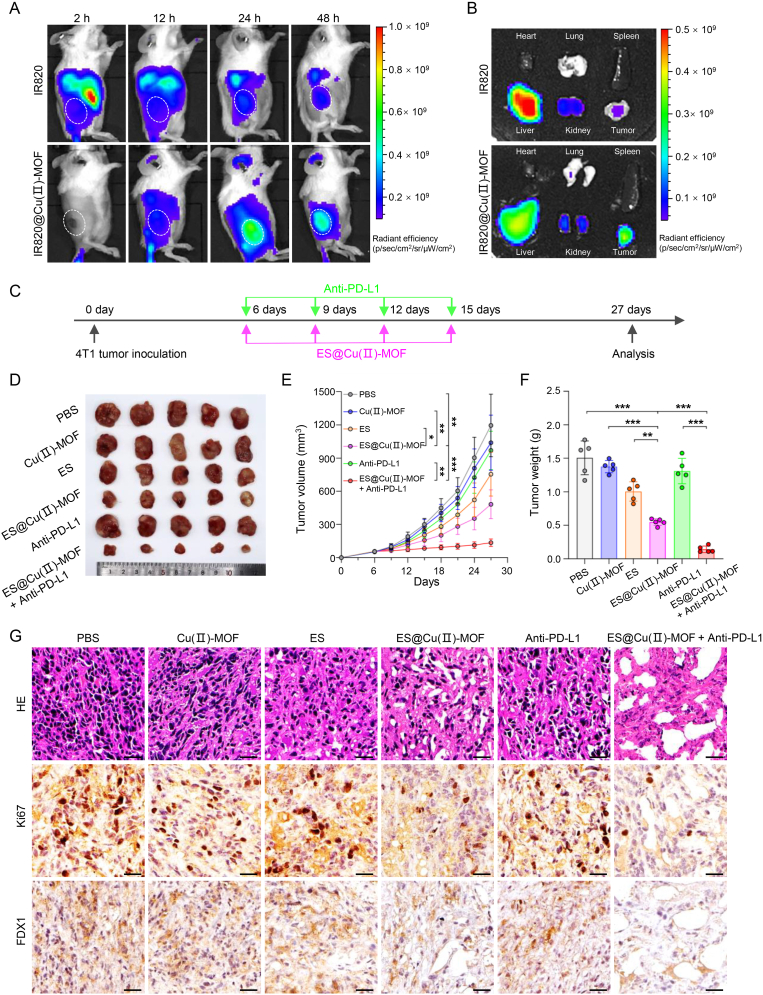
The antitumor effect of ES@Cu(Ⅱ)-MOF NPs combined with an Anti-PD-L1 antibody *in vivo*. A) IVIS imaging of subcutaneous tumor-bearing mice after intravenous injection of IR820 or IR820@Cu(Ⅱ)-MOF NPs. B) IVIS imaging of mouse tumors and major organs (heart, lung, kidney, liver, and spleen) collected at 48 h postinjection. C) Treatment schedule for the antitumor effect of ES@Cu(Ⅱ)-MOF + Anti-PD-L1 combination therapy *in vivo*. D-F) Photographs of tumors (D), growth curves (E), and tumor weights (F) of tumor tissues collected from 4T1 tumor-bearing mice after different treatments (n = 5). H) H&E staining and Ki-67 (nuclear positive) and FDX1 (cytoplasmic positive) IHC images of tumor tissues collected from 4T1 tumor-bearing mice following various treatments. Scale bars: 25 μm. The data are presented as the means ± SDs; ∗p < 0.05; ∗∗p < 0.01; ∗∗∗p < 0.001.

The *in vivo* biodistribution of ES@Cu(Ⅱ)-MOF NPs was determined with an *in vivo* optical imaging system (IVIS). The fluorescence signal corresponding to the new indocyanine green (IR820)-loaded Cu(Ⅱ)-MOF (IR820@Cu(Ⅱ)-MOF) in the tumor region gradually increased within 2 h and peaked at 24 h after intravenous injection. In addition, the continuous fluorescence signal remained strong at 48 h after IR820@Cu(Ⅱ)-MOF injection (Fig. 7A; Fig. S16). The tumor, heart, lung, liver, kidney, and spleen were subsequently collected for *ex vivo* analysis. Consistent with the *in vivo* fluorescence imaging results, the excised tumors from the IR820@Cu(Ⅱ)-MOF-injected mice exhibited a brighter fluorescence signals than the tumors from mice in the control group at 48 h postinjection. IR820@Cu(Ⅱ)-MOF was incapable of accumulating in the liver and kidney (Fig. 7B; Fig. S16) because of its unavoidable retention by the reticuloendothelial system [39]. Together, these results confirmed the sustained presence and efficient tumor-targeting ability of the ES@Cu(Ⅱ)-MOF NPs.

### 3.9 The antitumor ability of ES@Cu(Ⅱ)-MOF+Anti-PD-L1 combination therapy

Notably, 4T1 tumor cells expressed a very high level of PD-L1 (Fig. S17), which can directly bind to PD-1 on the surface of T cells to induce immune escape; thus, targeting the known immune checkpoint inhibitor PD-L1 could be a promising immunotherapy strategy [40,41]. Next, the antitumor efficacy of ES@Cu(Ⅱ)-MOF in combination with PD-L1-targeted therapy was further evaluated in 4T1 tumor-bearing mice. For this assay, 4T1 cells were subcutaneously injected into the right flanks of female BALB/c mice. Upon reaching a tumor volume of ∼60 mm³, the 4T1 tumor-bearing mice were randomly divided into six distinct groups. On days 6, 9, 12, and 15 after 4T1 tumor inoculation, the 4T1 tumor-bearing mice were treated with PBS, Cu(Ⅱ)-MOF NPs, ES, ES@Cu(Ⅱ)-MOF NPs, PD-L1-targeted therapy or ES@Cu(Ⅱ)-MOF + Anti-PD-L1 (Fig. 7C). The tumor size in the PBS group increased dramatically. In contrast, the ES and Cu(II)-MOF groups exhibited a minor reduction in tumor volume compared with the group treated with ES@Cu(II)-MOF. Notably, treatment with anti-PD-L1 therapy alone exhibited significantly lower antitumor efficacy in tumor-bearing mice. In contrast, the tumor size in the ES@Cu(Ⅱ)-MOF + Anti-PD-L1 group was markedly smaller than that in the ES@Cu(Ⅱ)-MOF or Anti-PD-L1 group, demonstrating the superior antitumor efficacy of the combination therapy (Fig. 7D and E). The marked changes in tumor weight were consistent with the changes visible in the digital images and the changes in the tumor volume in the different groups (Fig. 7D–F). Notably, ES@Cu(Ⅱ)-MOF treatment strongly inhibited tumor growth after combination with Anti-PD-L1, resulting in a tumor inhibition rate greater than 90 %, which was greater than that of the ES@Cu(Ⅱ)-MOF (63 %) and Anti-PD-L1(13 %) treatments (Fig. S18). Moreover, the survival of 4T1 tumor-bearing mice was significantly improved after ES@Cu(Ⅱ)-MOF + Anti-PD-L1 combination therapy (Fig. S19). Next, the marked antitumor efficacy of ES@Cu(Ⅱ)-MOF + Anti-PD-L1 was further confirmed by both hematoxylin and eosin (H&E) staining and Ki67 immunohistochemical (IHC) staining. After combined therapy with ES@Cu(II)-MOF and Anti-PD-L1, extensive vacuolation and significant necrosis were observed, in comparison to the moderate tissue damage observed in the group treated with ES@Cu(II)-MOF alone. Furthermore, the proliferation rate of cells in the group receiving the combined treatment was almost undetectable (Fig. 7G; Fig. S20), indicating that the ES@Cu(Ⅱ)-MOF + Anti-PD-L1 combination therapy resulted in severe tumor growth arrest. Finally, to determine whether cuproptosis is involved in the effects of the combination therapy *in vivo*, the expression of FDX1 was analyzed via IHC staining. In line with the *in vitro* results (Fig. 4), the tumors treated with ES@Cu(Ⅱ)-MOF + Anti-PD-L1 presented the greatest downregulation of FDX1 (Fig. 7G; Fig. S21). Overall, these results indicated that ES@Cu(Ⅱ)-MOF-triggered cuproptosis dramatically increased the efficacy of PD-L1-targeted therapeutic approaches against cancer.

### 3.10 ES@Cu(Ⅱ)-MOF + Anti-PD-L1 combination therapy remodeled the immunosuppressive TME

**Fig. 8:**
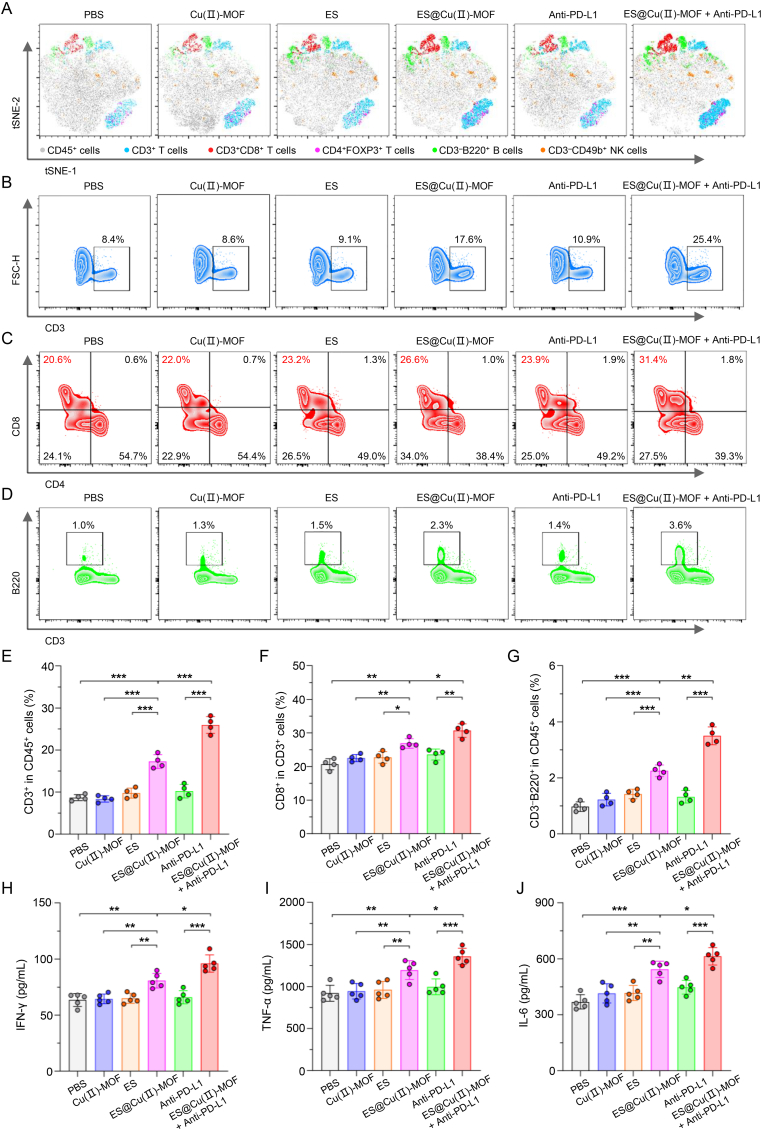
ES@Cu(Ⅱ)-MOF + Anti-PD-L1 combination therapy activated the antitumor immune response *in vivo*. A) T-SNE analyses of the distribution of TILs in tumor tissues collected from 4T1 tumor-bearing mice following various treatments. B) Flow cytometric analysis of CD3^+^ T lymphocytes in tumor tissues from 4T1 tumor-bearing mice following various treatments. C) Flow cytometric analysis of CD3^+^CD8^+^ CTLs in tumor tissues from 4T1 tumor-bearing mice following the indicated treatments. D) Flow cytometric analysis of CD3^-^B220^+^ B cells in tumor tissues from 4T1 tumor-bearing mice following the indicated treatments. E-G) Quantification of the proportions of CD3^+^, CD3^+^CD8^+^, and CD3^-^B220^+^ cells in 4T1 tumor-bearing mice following the indicated treatments (n = 4). H-J) Serum IFN-γ, TNF-α, and IL-6 levels in 4T1 tumor-bearing mice following the indicated treatments (n = 5). The data are presented as the means ± SDs; ∗p < 0.05; ∗∗p < 0.01; ∗∗∗p < 0.001.

Since cuproptosis in tumor cells is accompanied by the release of DAMPs [42], the amount of HMGB-1 released after combination therapy was measured via enzyme-linked immunosorbent assay (ELISA). The serum concentration of HMGB-1 markedly increased after ES@Cu(Ⅱ)-MOF treatment (Fig. S22), indicating that ES@Cu(Ⅱ)-MOF-triggered cuproptosis augmented the immune response in the TME of 4T1 tumors. To determine the effects of ES@Cu(II)-MOF and anti-PD-L1 combination therapy on the TME, tumor tissues were collected after the indicated treatments to investigate tumor-infiltrating lymphocytes (TILs) (Fig. S23). The distribution of TILs within the tumors was subsequently visualized via two-dimensional t-stochastic neighbor embedding (t-SNE) projection (Fig. 8A). ES@Cu(Ⅱ)-MOF + Anti-PD-L1 combination therapy significantly remodeled the immunosuppressive TME (Fig. 8A–G; Figs. S23–S26). Overall, the proportion of CD3^+^ cells in tumor tissues treated with ES@Cu(Ⅱ)-MOF + Anti-PD-L1 was obviously greater than that in the other groups (Fig. 8B and E). The number of CD8^+^ T lymphocytes, a major kind of cytotoxic T lymphocyte (CTL) that directly kills tumor cells [43,44], was greater in the tumors of the mice that received the ES@Cu(II)-MOF and Anti-PD-L1 combination therapy than in those that received either ES@Cu(II)-MOF or Anti-PD-L1 alone (Fig. 8C and F). Further IHC staining of CD8^+^ CTLs in tumors revealed a similar increasing trend (Fig. S24). In addition, the proportion of CD4^+^FOXP3^+^ regulatory T cells (Tregs), which play a critical role in immune escape [45], was noticeably reduced in the tumors of the mice after ES@Cu(Ⅱ)-MOF + Anti-PD-L1 combination therapy (Fig. S25). We further investigated the tumoricidal ability of infiltrated CD3^−^CD49b^+^ NK cells, another type of CTLs [43]. The results revealed more NK cells in the tumors of the mice after ES@Cu(Ⅱ)-MOF + Anti-PD-L1 combination therapy than in those of the other treatment groups (Fig. S26). Accordingly, the proportion of CD3^-^B220^+^ B cells, which are involved in antigen recognition and antibody production [46], was significantly increased in the tumors of the mice after ES@Cu(Ⅱ)-MOF + Anti-PD-L1 combination therapy (Fig. 8D and G). To determine the activation of the antitumor immune response, the levels of interferon-γ (IFN-γ), tumor necrosis factor-α (TNF-α), and interleukin 6 (IL-6) in the serum were further determined via ELISA. Compared with those in the other groups, the serum levels of IFN-γ, TNF-α, and IL-6 were markedly elevated in the mice after ES@Cu(Ⅱ)-MOF + Anti-PD-L1 combination therapy (Fig. 8H–J). Collectively, the above *in vivo* results were consistent with those of the *in vitro* study (Fig. 6; Fig. 8), further confirming that ES@Cu(Ⅱ)-MOF + Anti-PD-L1 could effectively enhance the antitumor immune response.

### 3.11 Hemolysis and biosafety *in vivo*

To thoroughly assess the biosafety profile of the ES@Cu(Ⅱ)-MOF + Anti-PD-L1 combination therapy, a comprehensive toxicological study was conducted to elucidate any potential material-related toxic effects. Therefore, we systematically collected blood and tissue samples from tumor-bearing mice following the indicated treatments for detailed examination. The histological integrity of the mouse organs was evaluated through H&E staining. Compared with the control, the ES@Cu(Ⅱ)-MOF + Anti-PD-L1 combination therapy did not result in any significant inflammatory area or pathological alterations in the vital tissues of 4T1 tumor-bearing mice (Fig. S27). Furthermore, blood biochemistry analysis demonstrated that the combination therapy did not lead to substantial hepatic or renal impairment (Fig. S28). In addition, the hemolysis assay results showed that the ES@Cu(Ⅱ)-MOF NPs had favorable biocompatibility and did not cause noticeable hemolysis even at high concentration (200 μg/mL) (Fig. S29). In addition, the ES@Cu(Ⅱ)-MOF NPs showed good stability in DMEM supplemented with 10 % FBS for 10 days, which further indicated the stability of ES@Cu(Ⅱ)-MOF NPs in biological applications (Fig. S30). Finally, the body weight remained unaltered in the combination therapy group relative to the control group (Fig. S31). Collectively, these findings demonstrated the favorable biocompatibility and safety profile of the ES@Cu(Ⅱ)-MOF + Anti-PD-L1 combination therapy for *in vivo* anticancer applications.

## 4 Conclusions

In summary, an ES@Cu(Ⅱ)-MOF nanocomposite with enzyme-mimicking activity was successfully synthesized for use as an effective cuproptosis inducer. With a low pH in the lysosomes of 4T1 cells, the structure of ES@Cu(Ⅱ)-MOF NPs was degraded to release Cu^2+^ and ES simultaneously, resulting in a stronger cytotoxic effect on cancer cells. Importantly, ES@Cu(Ⅱ)-MOF nanozymes with outstanding enzyme-mimicking activities (POD-like and GSH-Px-like) catalyzed the generation of toxic •OH and consumed endogenous GSH, thus further sensitizing cells to cuproptosis. Mechanistically, the intracellular accumulation of Cu^+^ and ES contributed to the aggregation of lipoylated DLAT and the downregulation of Fe‒S cluster proteins, ultimately triggering cuproptosis. Moreover, the ES@Cu(Ⅱ)-MOF nanozymes effectively accumulated in the tumor region, leading to significant suppression of tumor growth in the 4T1 mouse model. Notably, ES@Cu(Ⅱ)-MOF effectively triggered robust ICD, which promoted an antitumor immune response and subsequently improved the therapeutic effectiveness of immune checkpoint blockade (ICB) treatments. ES@Cu(Ⅱ)-MOF nanozymes combined with an anti-PD-L1 antibody successfully converted “cold” tumors into “hot” tumors, thereby achieving excellent antitumor effects and exhibiting good biocompatibility. Collectively, our unique nanoplatform design provides a new approach for integrating Cu-based nanozymes to induce cuproptosis and improve the outcomes of combined immunotherapy (Fig. 1).

## CRediT authorship contribution statement

**Xufeng Lu:** Writing – review & editing, Writing – original draft, Investigation, Funding acquisition. **Wenhai Deng:** Investigation, Funding acquisition. **Shuaibin Wang:** Validation, Investigation. **Shengsheng Zhao:** Visualization. **Bingzi Zhu:** Methodology. **Binglong Bai:** Formal analysis. **Yiwen Mao:** Resources. **Ji Lin:** Data curation. **Yongdong Yi:** Visualization. **Zuoliang Xie:** Resources, Funding acquisition. **Xiang Wang:** Software. **Yongyong Lu:** Funding acquisition. **Xiufeng Huang:** Resources. **Tao You:** Resources. **Xiaolei Chen:** Conceptualization. **Weijian Sun:** Supervision, Funding acquisition. **Xian Shen:** Writing – review & editing, Supervision, Project administration, Funding acquisition.

## Declaration of competing interest

The authors declare that they have no known competing financial interests or personal relationships that could have appeared to influence the work reported in this paper.

## Data Availability

Data will be made available on request.
