# Supplementary material for: PEGylated Elesclomol@Cu(Ⅱ)-based Metal‒organic framework with effective nanozyme performance and cuproptosis induction efficacy for enhanced PD-L1-based immunotherapy
Source: Mater Today Bio. 2024 Oct 28;29:101317. doi: 10.1016/j.mtbio.2024.101317 (PMC11565527; doi:10.1016/j.mtbio.2024.101317)
Supplement: Multimedia component 1 [file mmc1.docx]

**PEGylated Elesclomol@Cu(Ⅱ)-based Metal‒organic Framework with Effective Nanozyme Performance and Cuproptosis Induction Efficacy for Enhanced PD-L1-based Immunotherapy**

**Xufeng Lu^1,2,3,*^, Wenhai Deng^4,*^, Shuaibin Wang^1,*^, Shengsheng Zhao^1^, Bingzi Zhu^1^, Binglong Bai^1^, Yiwen Mao^5^, Ji Lin^2^, Yongdong Yi^1^, Zuoliang Xie^3^, Xiang Wang^1,2^, Yongyong Lu**^6^**, Xiufeng Huang^1^, Tao You^1^, Xiaolei Chen^2,#^, Weijian Sun^1,2,#^, Xian Shen^1,2,#^**

^1^ Zhejiang International Scientific and Technological Cooperation Base of Translational Cancer Research, Department of Gastrointestinal Surgery, The Second Affiliated Hospital and Yuying Children's Hospital of Wenzhou Medical University, Wenzhou, Zhejiang, 325000, China.

^2^ Zhejiang Key Laboratory of Intelligent Cancer Biomarker Discovery and Translation, Department of Gastrointestinal Surgery, The First Affiliated Hospital, Wenzhou Medical University, Wenzhou, Zhejiang, 325000, China.

^3^ Research Center of Basic Medicine, The Second Affiliated Hospital and Yuying Children's Hospital of Wenzhou Medical University, Wenzhou, Zhejiang, 325000, China.

^4^ Oujiang Laboratory (Zhejiang Lab for Regenerative Medicine, Vision, and Brain Health), School of Laboratory Medicine and Life Sciences, Wenzhou Medical University, Wenzhou, Zhejiang, 325000, China

^5^ Department of Breast Surgery, The Second Affiliated Hospital and Yuying Children's Hospital of Wenzhou Medical University, Wenzhou, Zhejiang, 325000, China.

^6^ Department of Urology, The First Affiliated Hospital of Wenzhou Medical University, Wenzhou, Zhejiang, 325000, China.

**^*^Co-first authors.**

**^#^Corresponding author:**

chenxiaolei@wmu.edu.cn (XL. Chen);

fame198288@126.com (WJ. Sun);

shenxian1120@126.com (X. Shen).

**Supporting information**

**Materials.**

Cu(NO_3_)_2_·3H_2_O, DSPE-PEG_2000_-NH_2_, FITC, H_2_O_2_, DTNB, TMB, and IR820 were obtained from Shanghai Aladdin Biochemical Technology Co., Ltd. BTC was obtained from Sigma Aldrich. Elesclomol (Cat: HY-12040) was purchased from MedChemExpress. PBS, FBS, DMEM, RPMI 1640 medium, 0.25% trypsin-EDTA and penicillin/streptomycin were obtained from Gibco. Annexin V-FITC/PI Apoptosis Detection Kit (Cat: 556547) was purchased from BD Biosciences. FDX1 antibody (Cat: ab108257) was purchased from Abcam. DLD antibody (Cat: 16431-1-AP) was purchased from Proteintech. DLAT antibody (Cat: 9661), β-Actin antibody (Cat: 3700), anti-mouse IgG, HRP-linked antibody (Cat: 7076), and anti-rabbit IgG, HRP-linked antibody (Cat: 7074) were purchased from Cell Signaling Technology, Inc. Cell Counting Kit-8 (Cat: CK04), Cytotoxicity LDH Assay Kit (Cat: CK12), and ATP Assay Kit-Luminescence (Cat: CK18) were obtained from Dojindo Laboratories. *InVivoMAb* anti-mouse PD-L1 (Cat: BE0101) was obtained from Bio X Cell. TruStain FcX™ PLUS antibody (Cat: 156604), PE/Cyanine5 anti-mouse CD45 antibody (Cat: 103110), PE anti-mouse CD3 antibody (Cat: 100206), FITC anti-mouse CD4 antibody (Cat: 100406), APC/Cyanine7 anti-mouse CD8a antibody (Cat: 100714), B220-Brilliant Violet 785 (Cat: 103246), and APC anti-mouse CD49b antibody (Cat: 108910) were obtained from BioLegend, Inc. LIVE/DEAD Fixable Dead Cell Stain Kit (Cat: L34955), PE-Cyanine7 FOXP3 Monoclonal Antibody (Cat: 25-5773-82), Ki-67 Monoclonal Antibody (Cat: 14-5698-82), DAPI (Cat: D3571), Hoechst 33342 (Cat: H1399), Goat anti-Mouse IgG (H+L) Cross-Adsorbed Secondary Antibody, Alexa Fluor™ 488 (Cat: A-11001) were obtained from Invitrogen. BCA Protein Quantification Kit (Cat: 20201) and Precast Protein Plus Gel (4-12%, 15 wells) (Cat: 36255) were obtained from Yeasen Biotechnology Co., Ltd (Shanghai). Total Glutathione Assay Kit, Calcein-AM/PI Live/Dead Viability Assay Kit, RIPA, Protease Inhibitor Cocktail, LysoTracker Red, MitoTracker Red CMXRos, Crystal violet, DCFH-DA and Dil were purchased from Shanghai Beyotime Biotechnology Co., Ltd. ELISA kits were obtained from Jiangsu Meimian Industrial Co., Ltd.


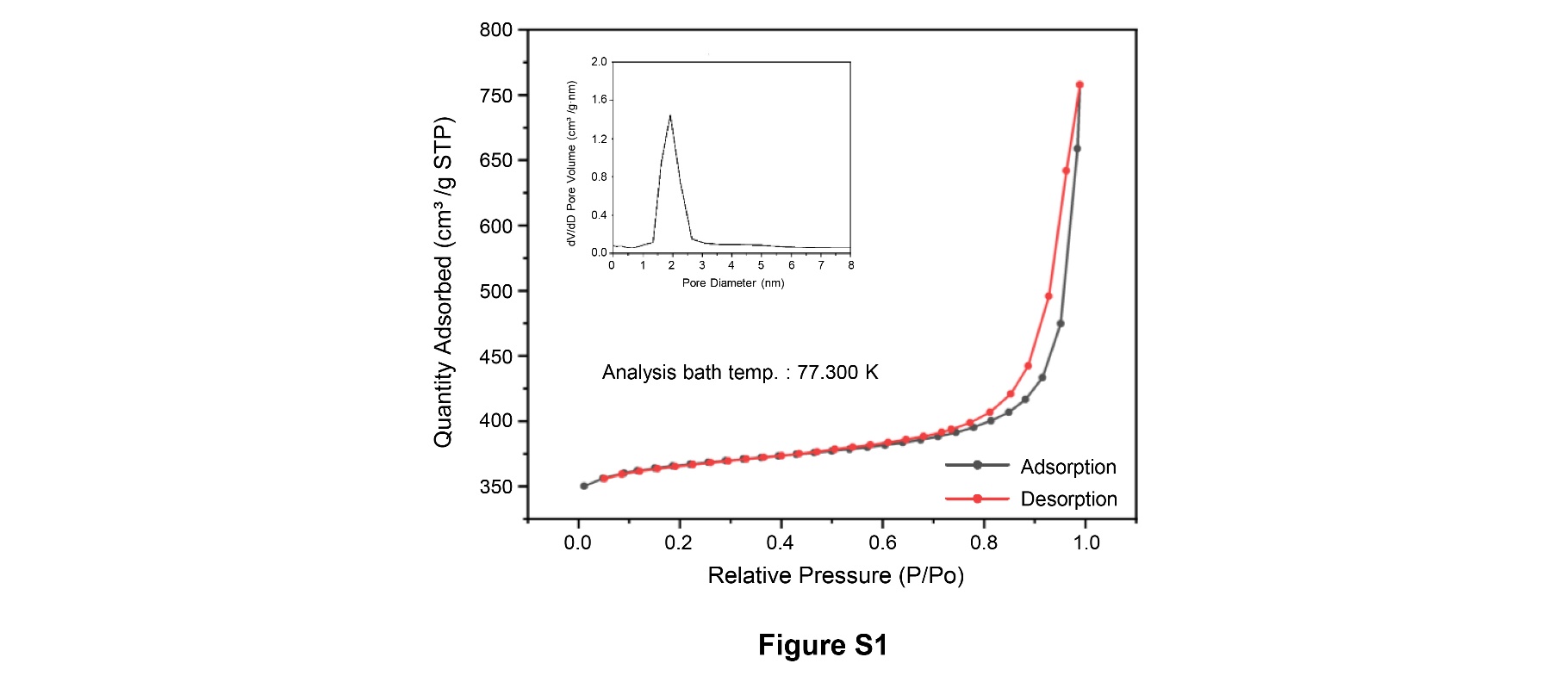


**Figure S1.** N_2_ adsorption-desorption isotherms of the Cu(Ⅱ)-MOF NPs.


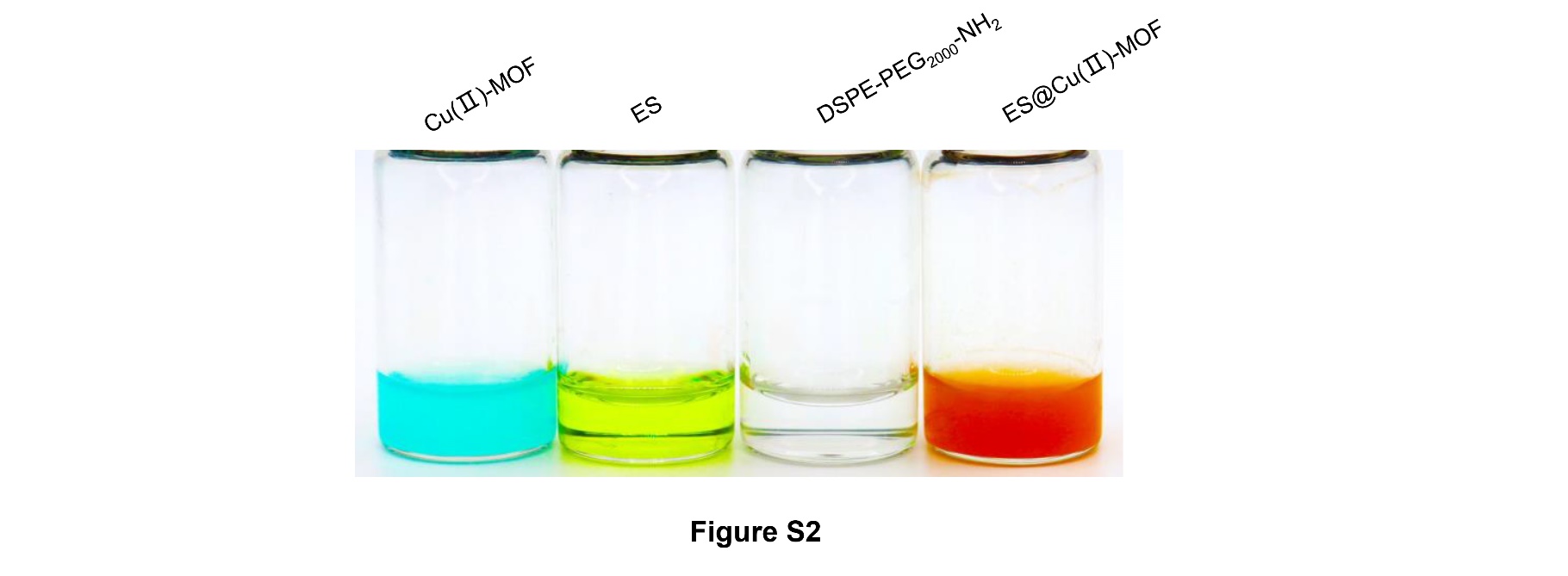


**Figure S2.** Photograph of synthesized ES@Cu(Ⅱ)-MOF NPs.


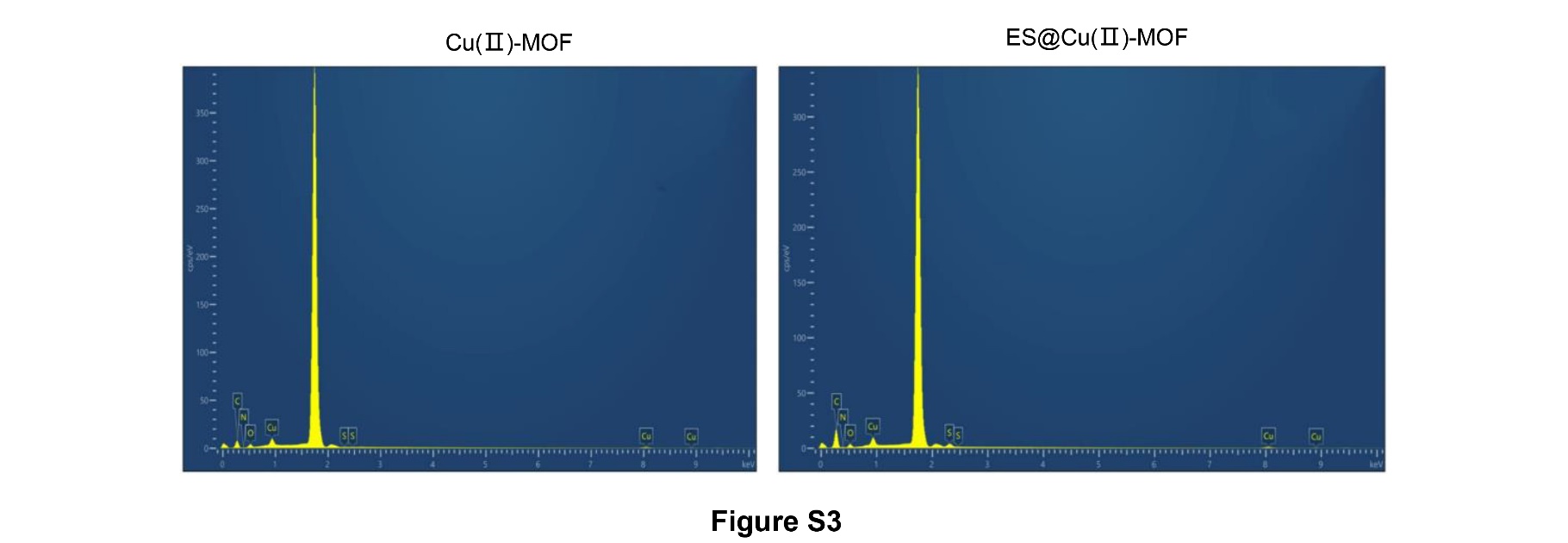


**Figure S3.** Sum energy dispersive spectroscopy (EDS) spectra of the Cu(Ⅱ)-MOF NPs and ES@Cu(Ⅱ)-MOF NPs, respectively.


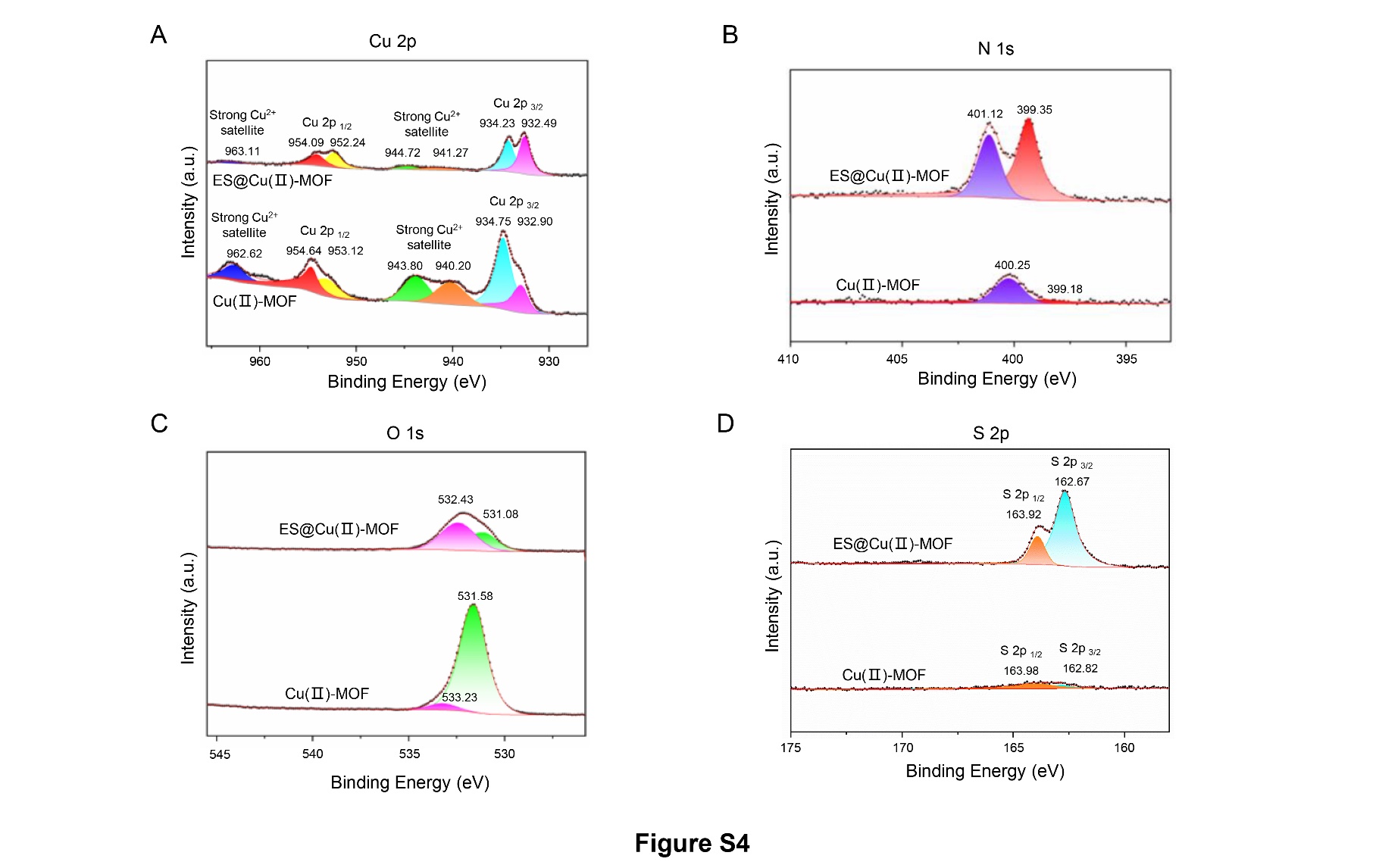


**Figure S4.** High-resolution XPS spectra of the Cu, N, O, and S orbitals of the Cu(Ⅱ)-MOF NPs and ES@Cu(Ⅱ)-MOF NPs, respectively. The chemical formula of ES is C_19_H_20_N_4_O_2_S_2_.


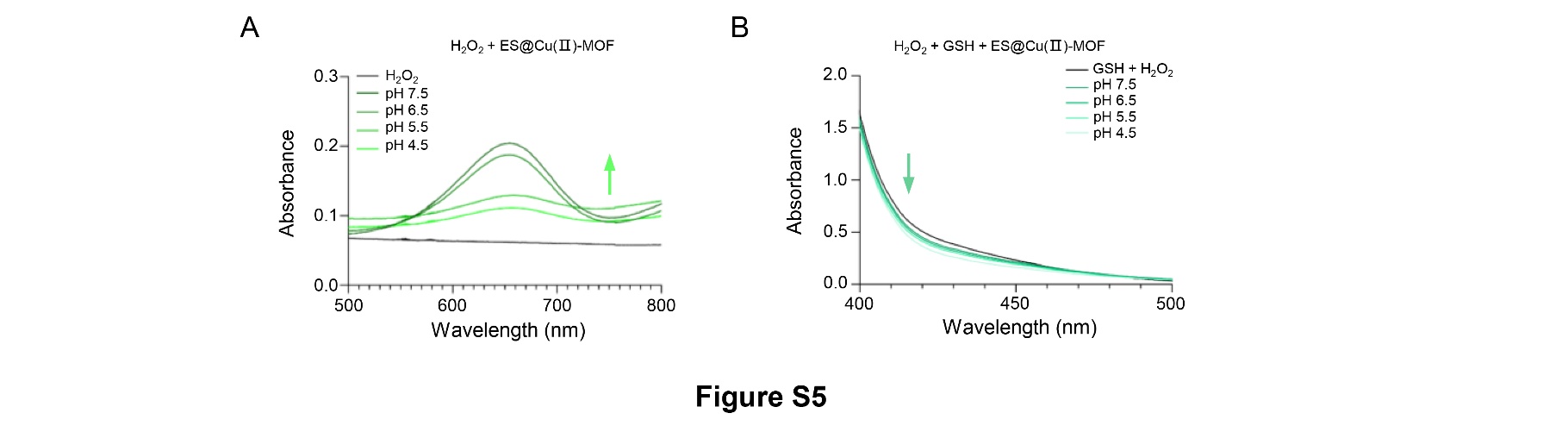


**Figure S5.** POD-like and GSH-Px-like activities of the ES@Cu(Ⅱ)-MOF NPs in different pH solutions.


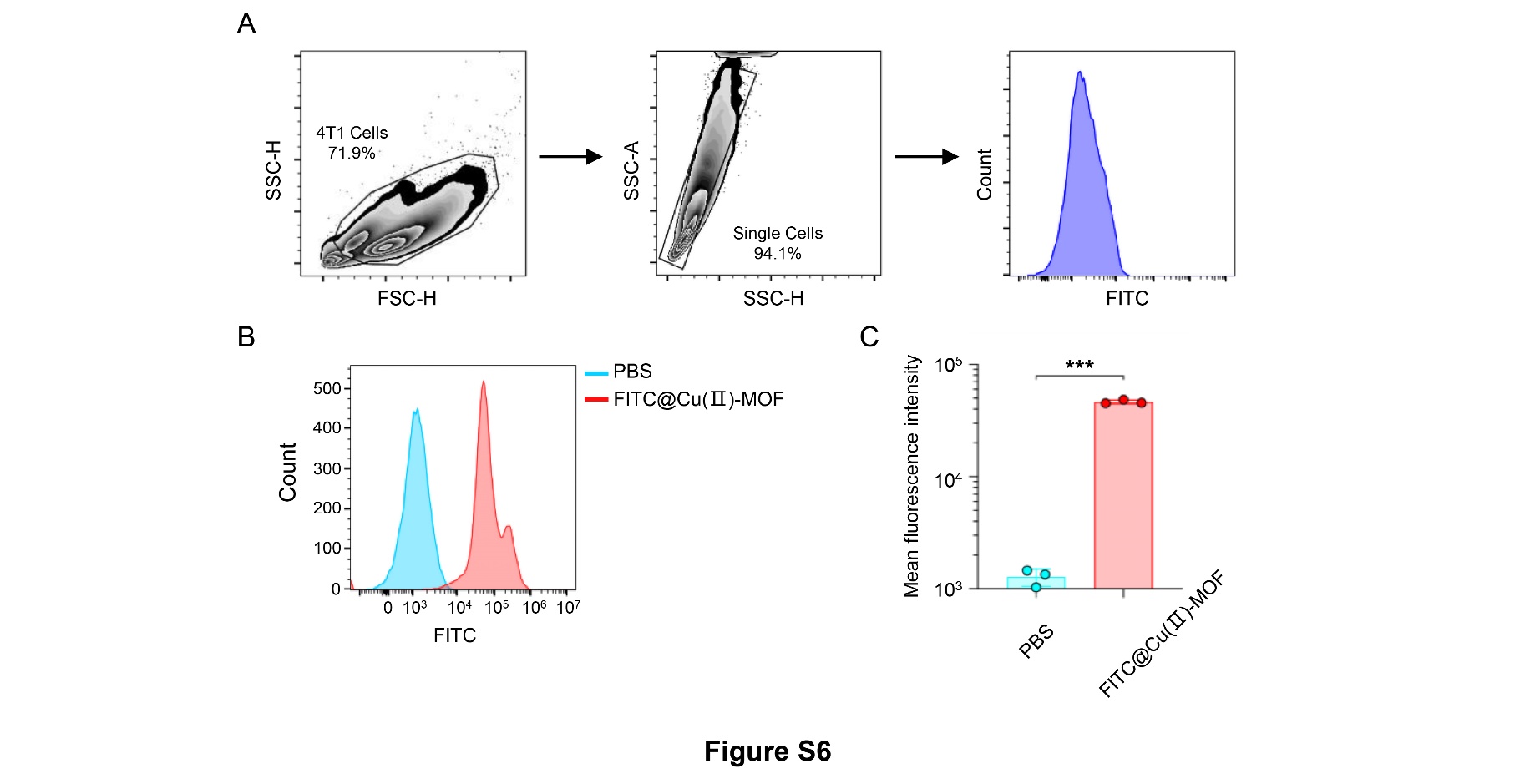


**Figure S6.** The cellular uptake of FITC@Cu(Ⅱ)-MOF NPs.

(A) Gate strategy for 4T1 cells following the indicated treatments.

(B) Flow cytometric analysis of 4T1 cells treated with PBS or FITC@Cu(Ⅱ)-MOF NPs.

(C) Quantification of the FITC intensity in 4T1 cells after the indicated treatment (n = 3). The data are presented as the means ± SDs; ***p < 0.001.


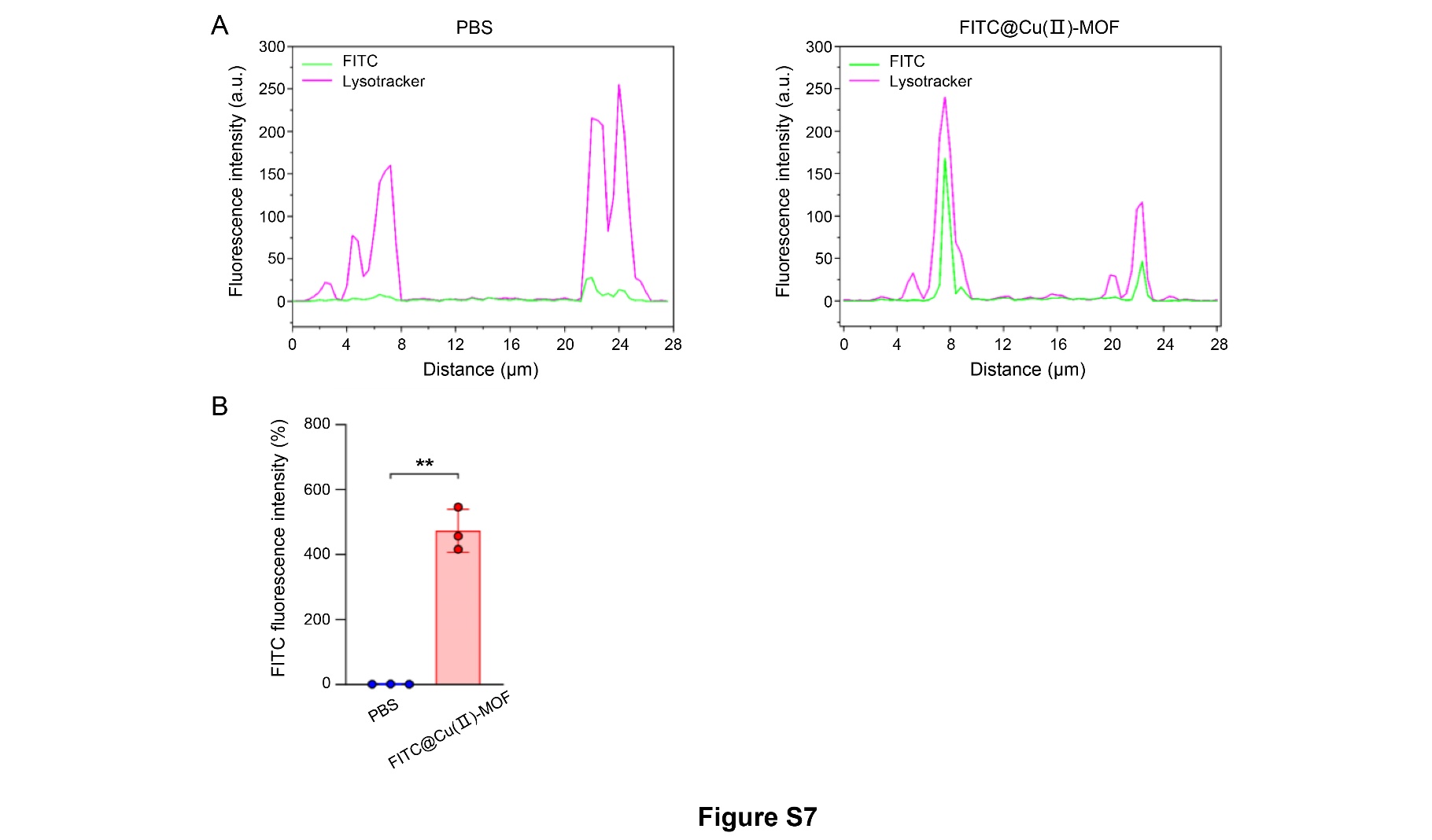


**Figure S7.** The fluorescence intensities of the FITC@Cu(Ⅱ)-MOF (green) and lysosome (pink) signals across the regions indicated with white arrows were analyzed and shown in line plots (Figure 4A). n = 3. The data are presented as the means ± SDs; **p < 0.01.


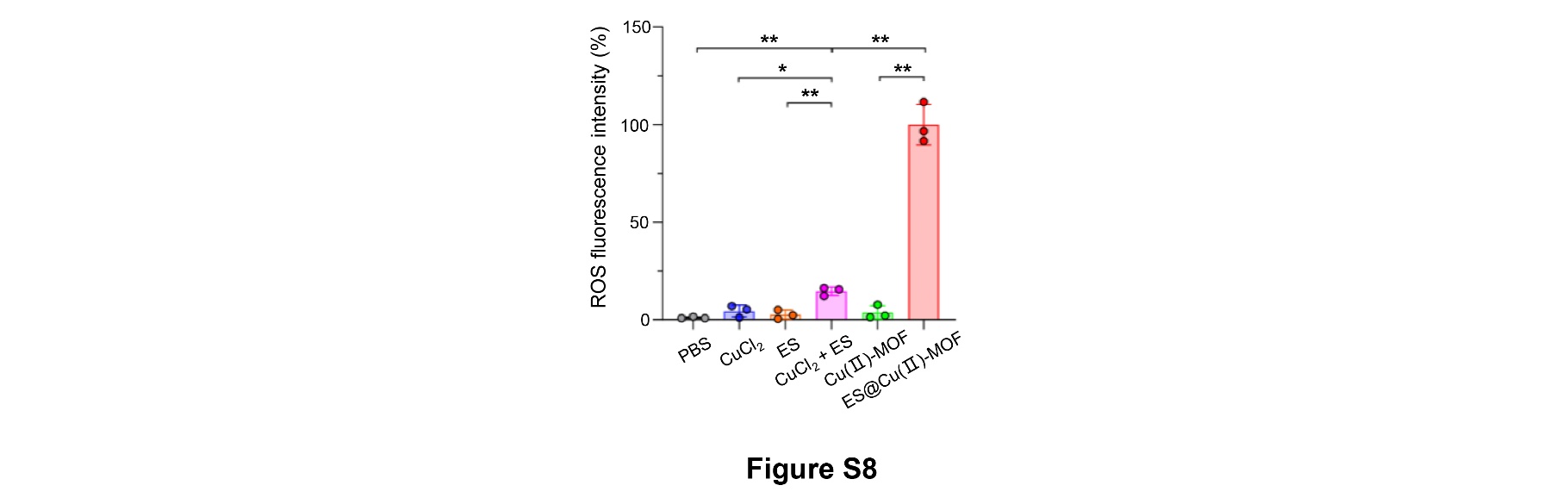


**Figure S8.** The ROS fluorescence intensity in 4T1 cells after the indicated treatments was quantified (n = 3). The data are presented as the means ± SDs; *p < 0.05; **p < 0.01.


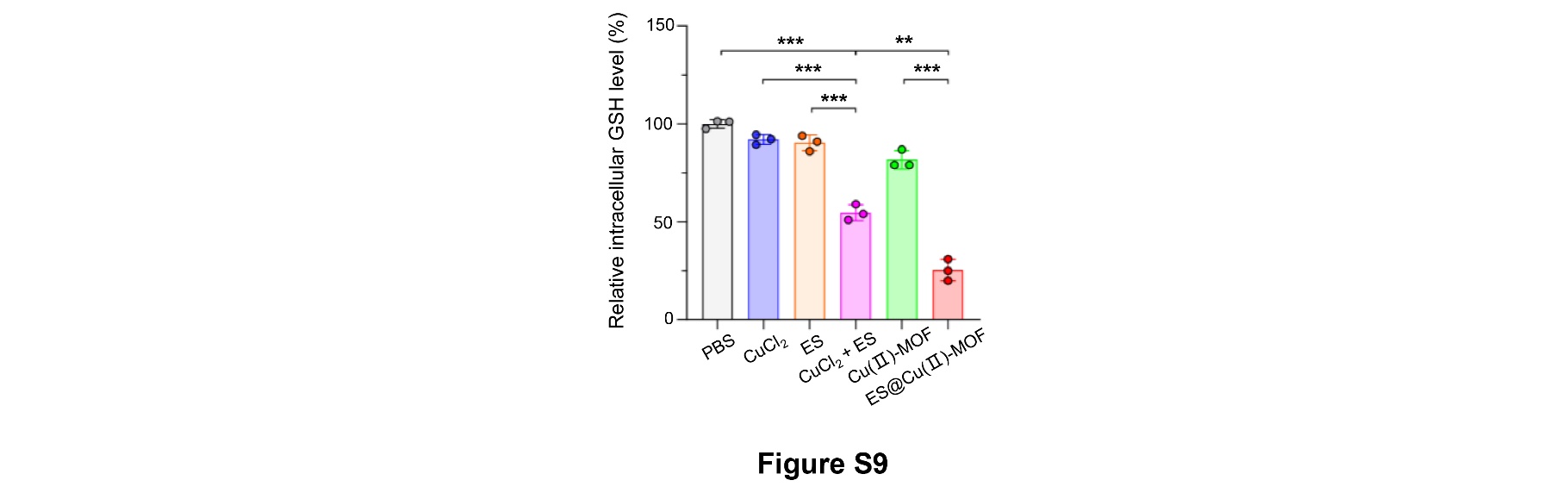


**Figure S9.** Intracellular GSH levels in 4T1 cells after the indicated treatments (n = 3).

The data are presented as the means ± SDs; **p < 0.01; ***p < 0.001.


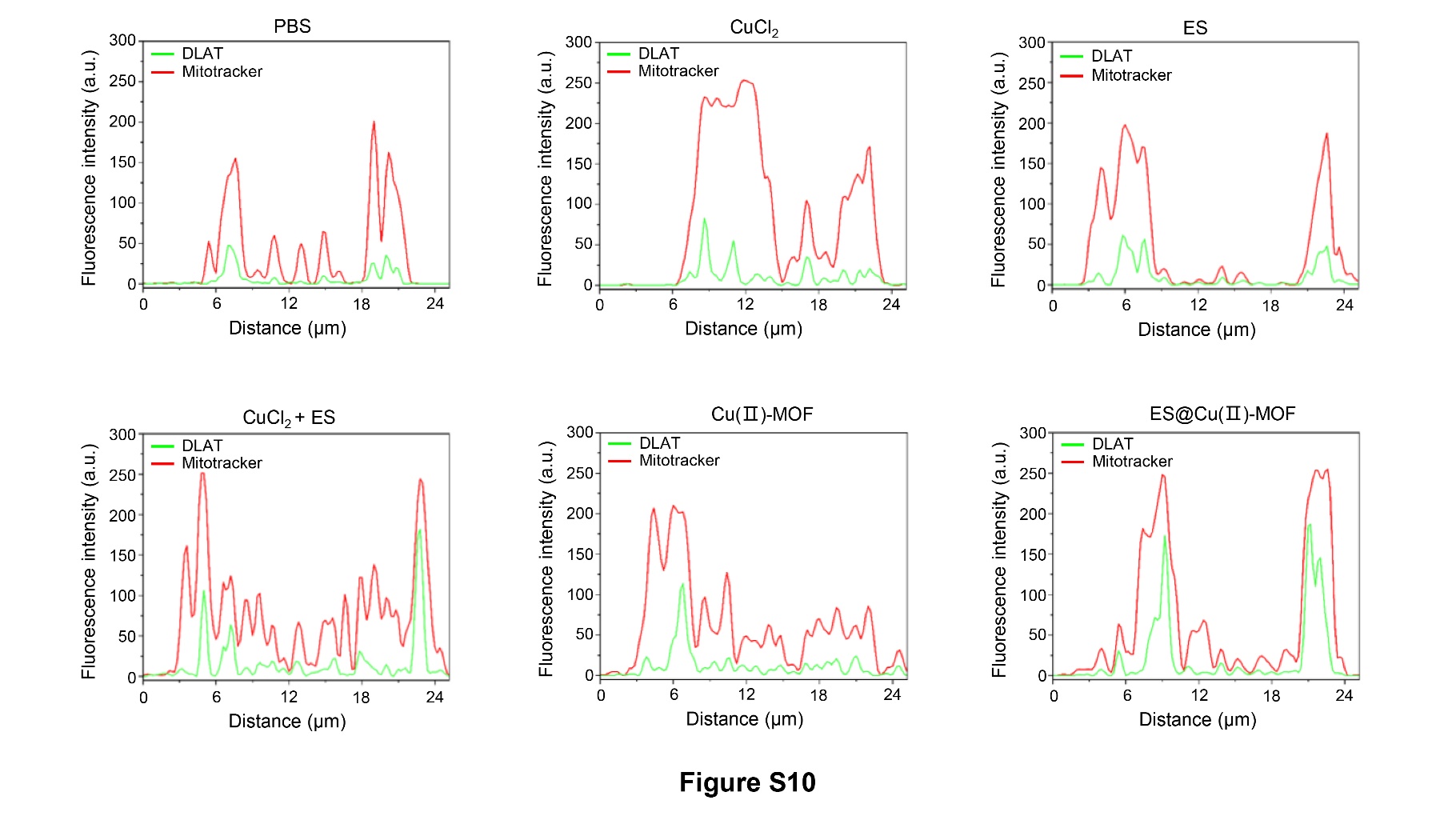


**Figure S10.** The fluorescence intensities of DLAT foci (green) and mitochondrial (red) signals across the regions indicated with white arrows were analyzed and shown in line plots (Figure 4D).


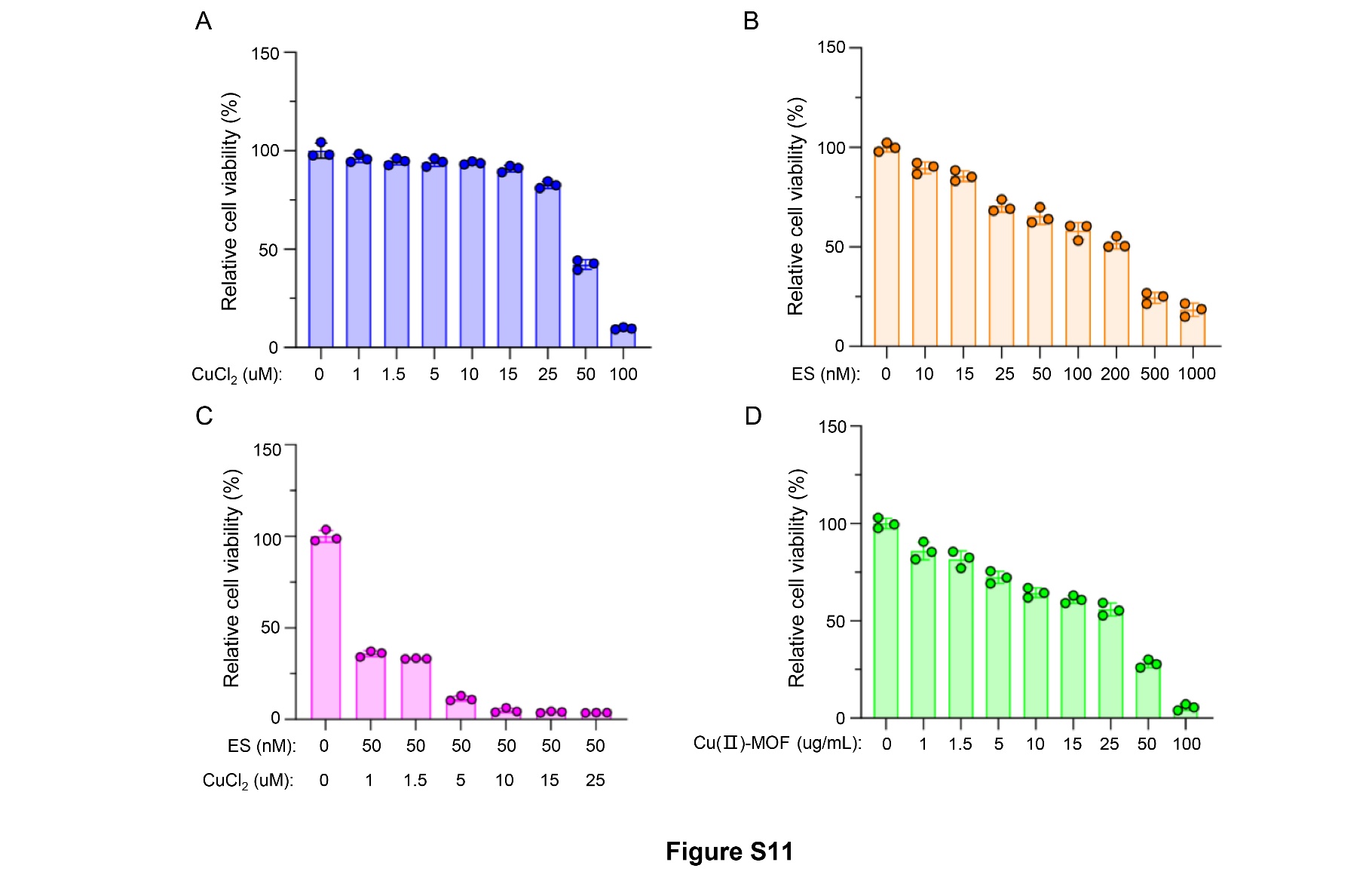


**Figure S11.** Relative viabilities of 4T1 cells following 24 h of incubation with CuCl_2_ (A), ES (B), CuCl_2_ + ES (C), or Cu(Ⅱ)-MOF NPs (D), respectively (n = 3).


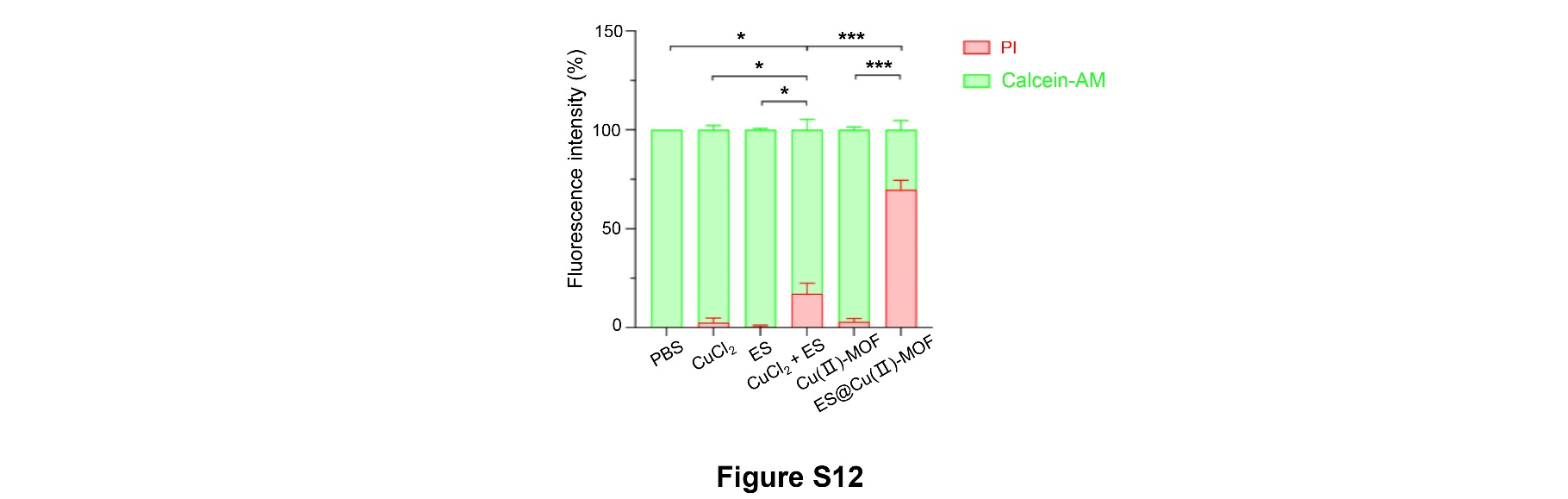


**Figure S12.** Quantification of calcein-AM^+^ and PI^+^ 4T1 cells after the indicated treatments (n = 3). The data are presented as the means ± SDs; *p < 0.05; ***p < 0.001.


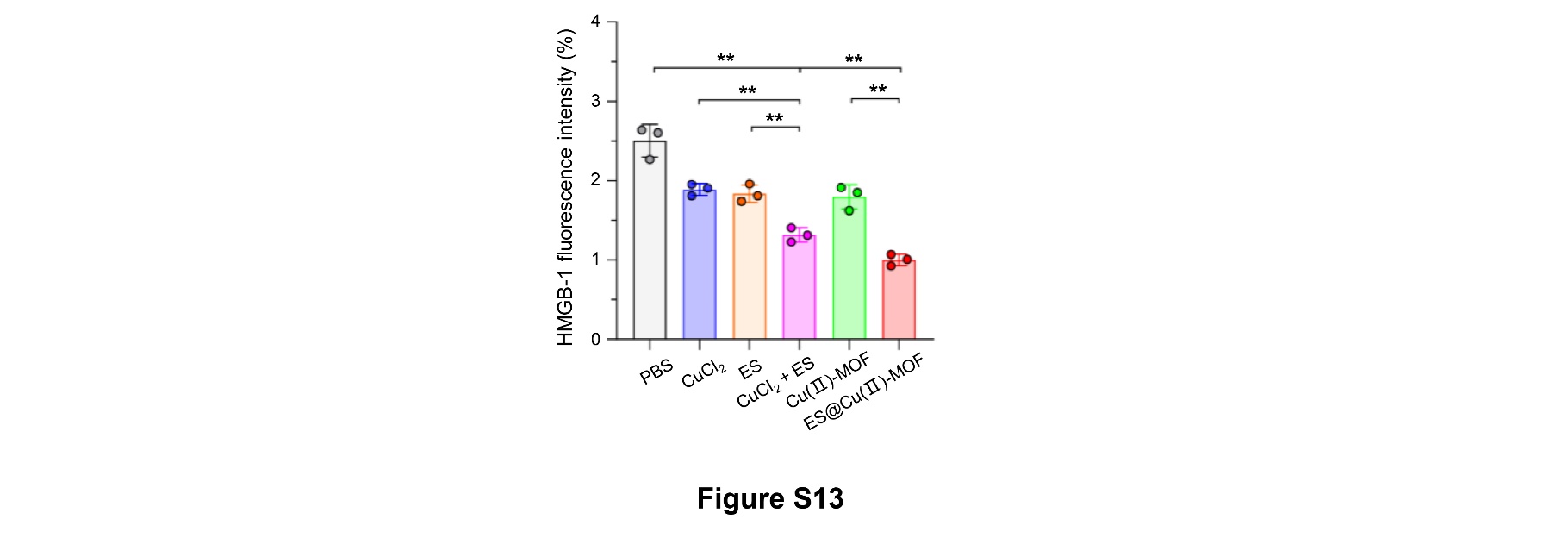


**Figure S13.** Quantification of HMGB-1 fluorescence intensity in 4T1 cells after the indicated treatments (n = 3). The data are presented as the means ± SDs; **p < 0.01.


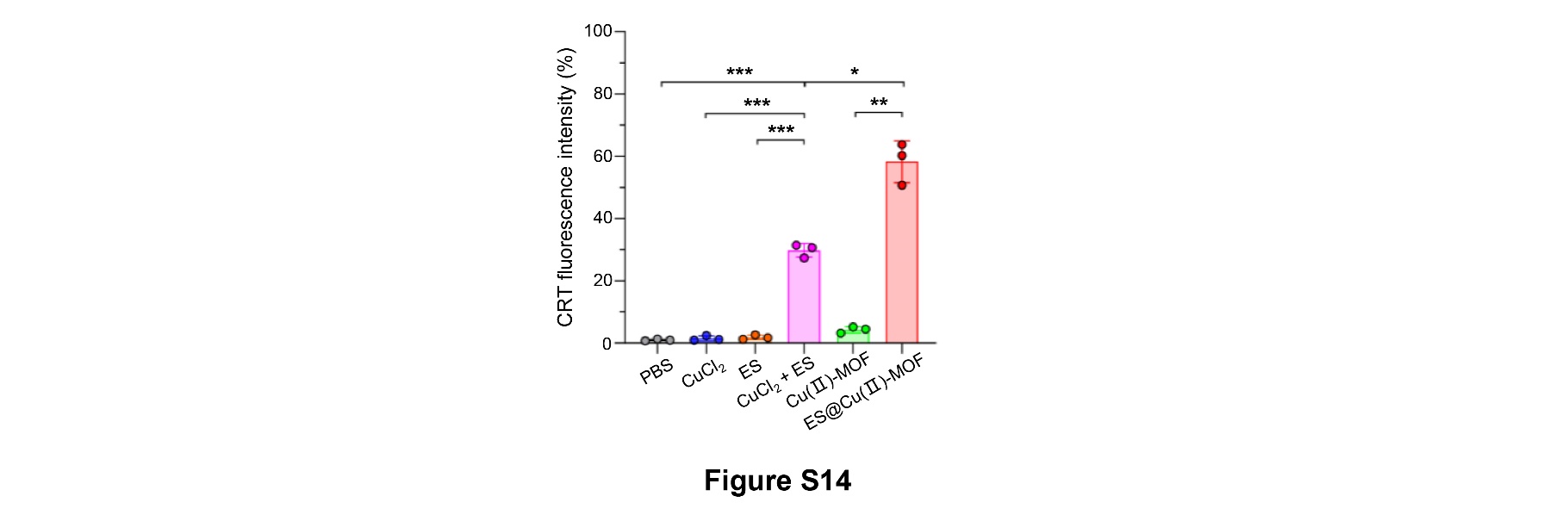


**Figure S14.** Quantification of CRT fluorescence intensity in 4T1 cells after the indicated treatments (n = 3). The data are presented as the means ± SDs; *p < 0.05; **p < 0.01; ***p < 0.001.


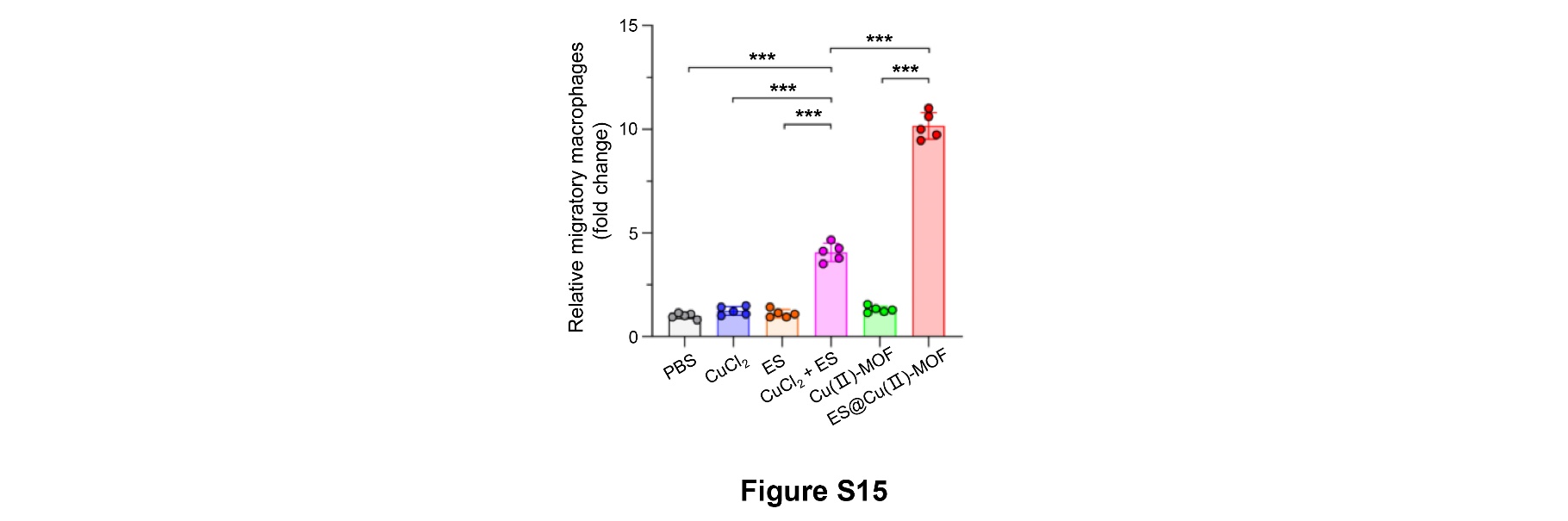


**Figure S15.** The chemotaxis rate of RAW264.7 macrophages stimulated with the indicated conditioned medium (n = 5). The data are presented as the means ± SDs; ***p < 0.001.


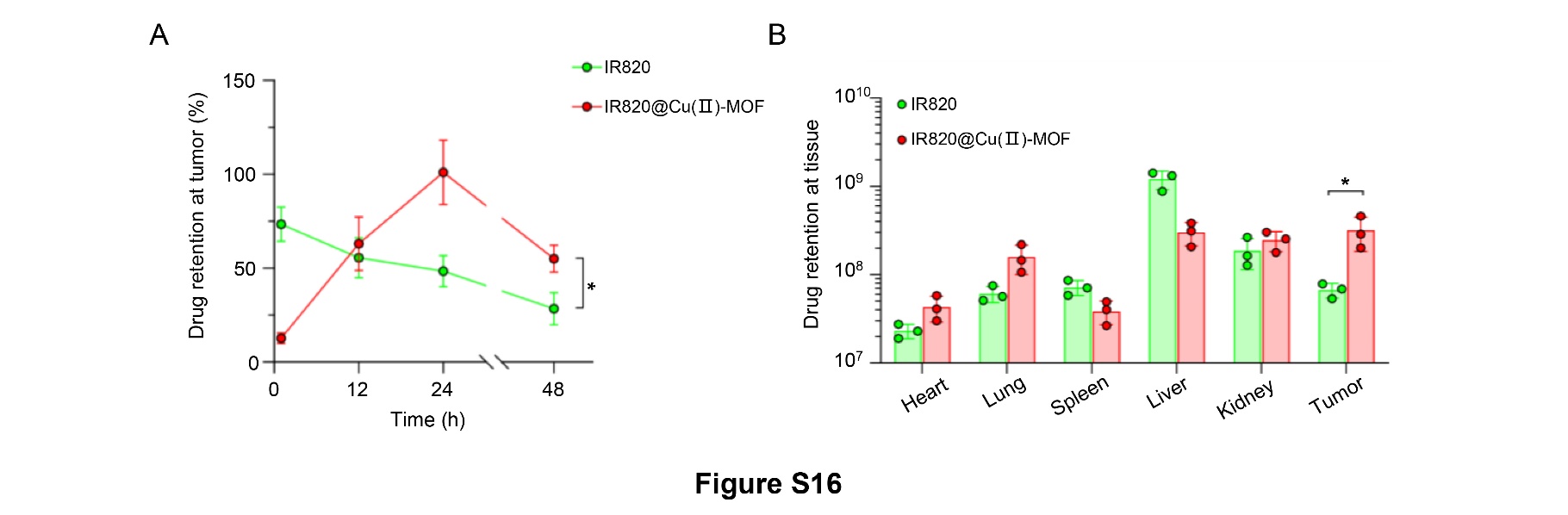


**Figure S16.** Fluorescence intensity in subcutaneous tumors and *ex vivo* major organs analyzed by IVIS imaging.

(A) Quantification of fluorescence intensity in subcutaneous 4T1 tumor-bearing mice after intravenous injection (n = 3).

(B) Quantification of fluorescence intensity in *ex vivo* mouse tumors and major organs (heart, lung, kidney, liver, and spleen) collected at 48 h postinjection (n = 3). The data are presented as the means ± SDs; *p < 0.05.


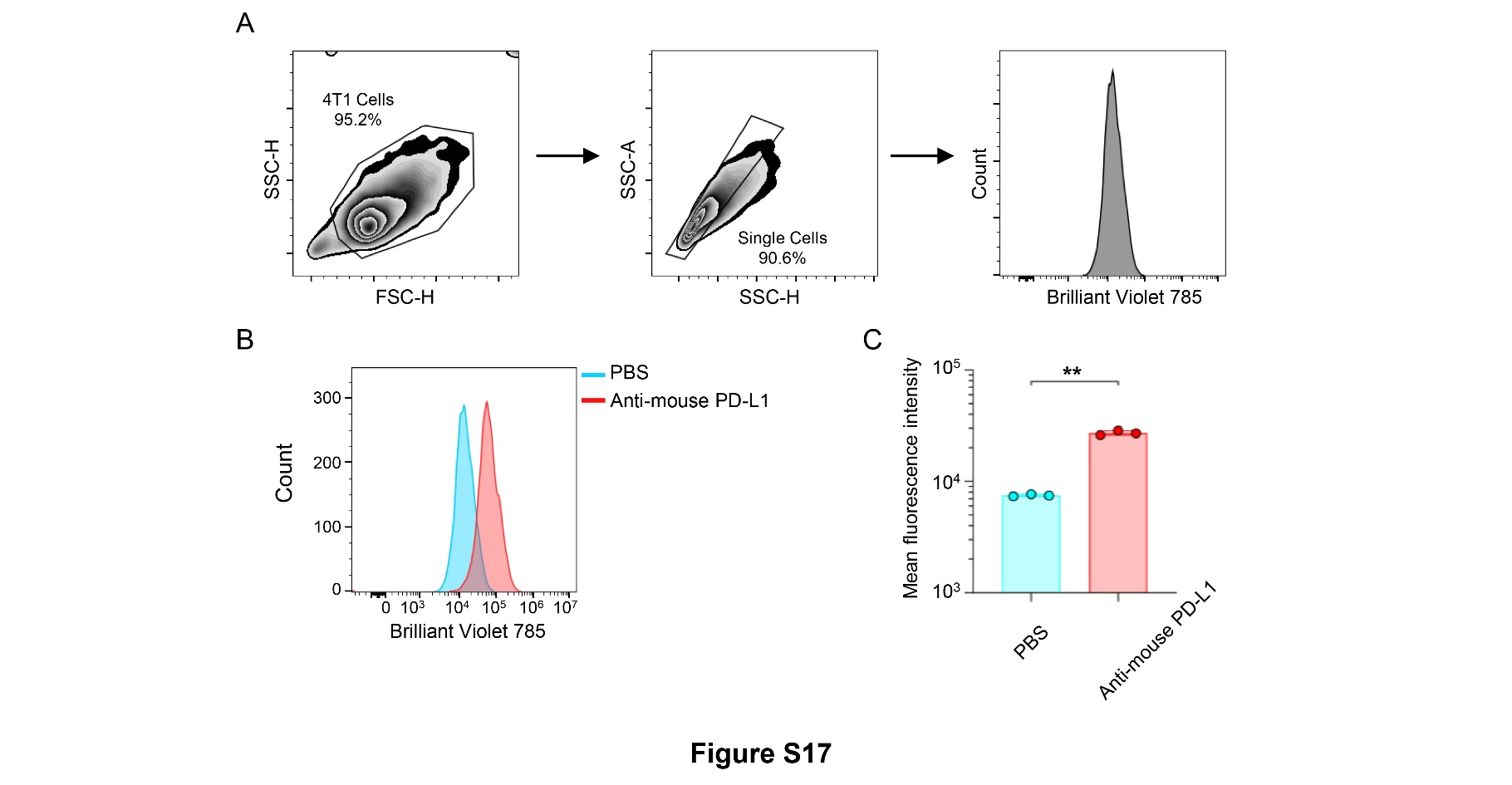


**Figure S17.** PD-L1 expression level in 4T1 cells.

(A) Gate strategy for analyzing the expression level of PD-L1 in 4T1 cells via the Brilliant Violet 785-conjugated anti-mouse PD-L1 antibody.

(B) Flow cytometric analysis of 4T1 cells treated with PBS or Brilliant Violet 785-conjugated anti-mouse PD-L1 antibody.

(C) Quantification of the mean fluorescence intensity of PD-L1 expression in 4T1 cells (n = 3). The data are presented as the means ± SDs; **p < 0.01.


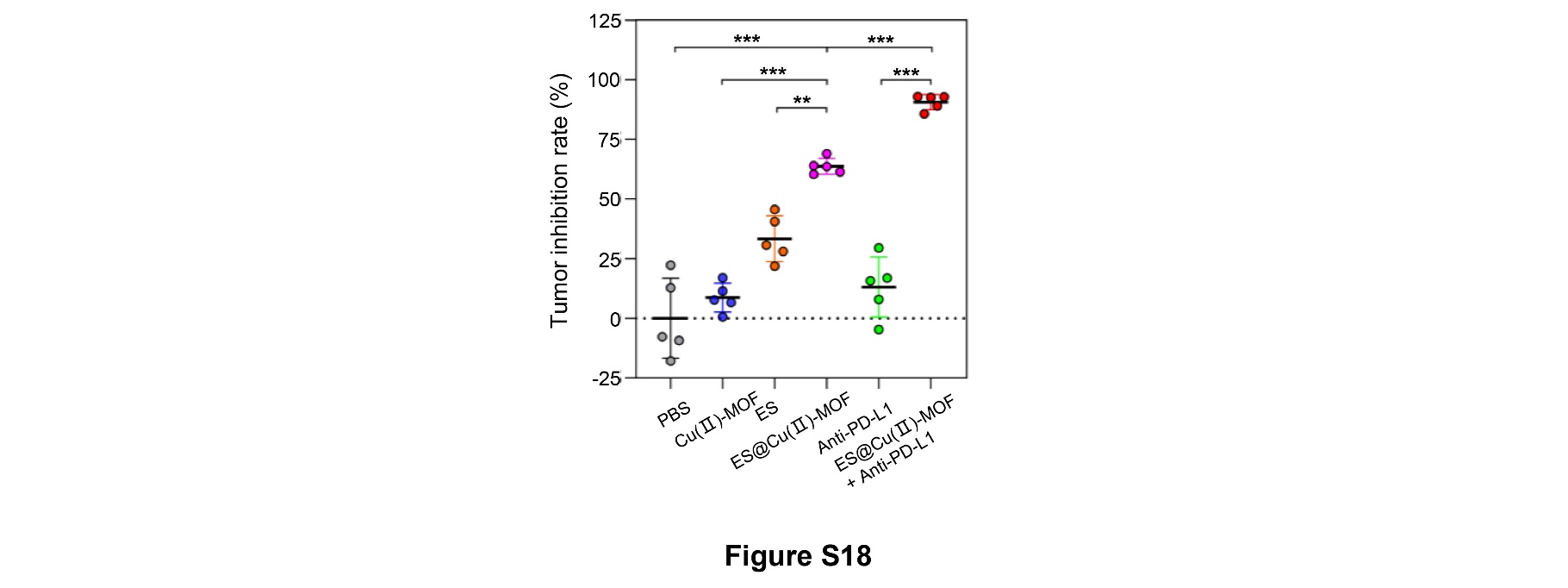


**Figure S18.** Tumor inhibition rates of mice subjected to different treatments. The tumor inhibition rate (%) = (1- W_Treat_/W_Control_) × 100%, where W_Treat_ represented the tumor weight in the treatment group, W_Control_ represented the tumor weight in the control group (PBS).


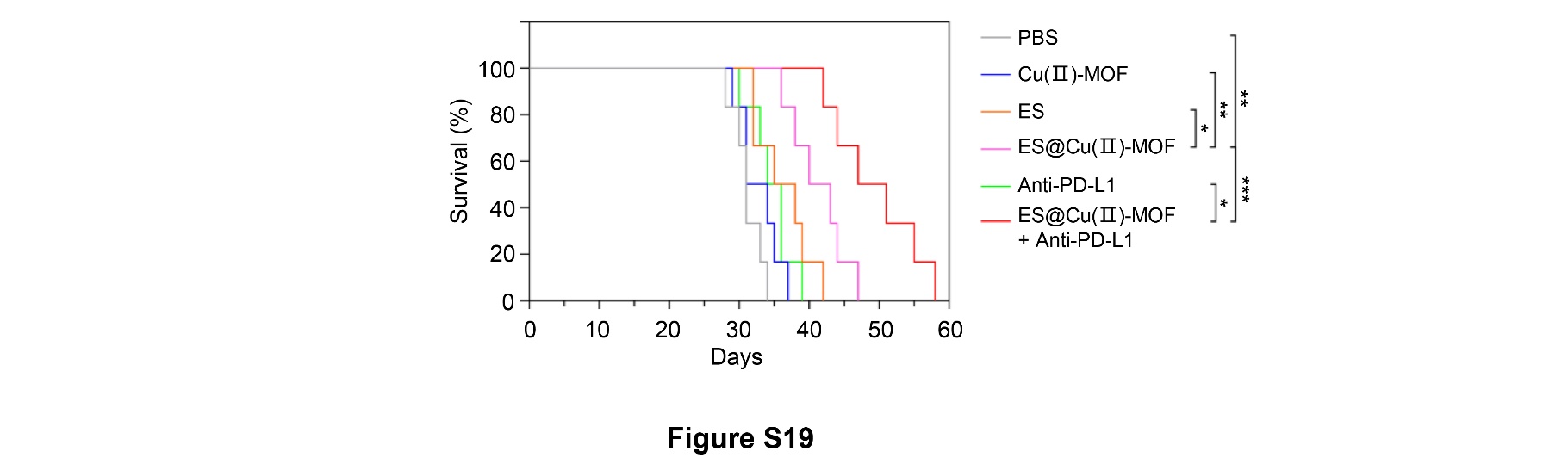


**Figure S19.** Survival curve of 4T1 tumor-bearing mice after the indicated treatments (n = 6). The survival rate was determined via Kaplan‒Meier analysis followed by the log-rank test; *p < 0.05; **p < 0.01; ***p < 0.001.


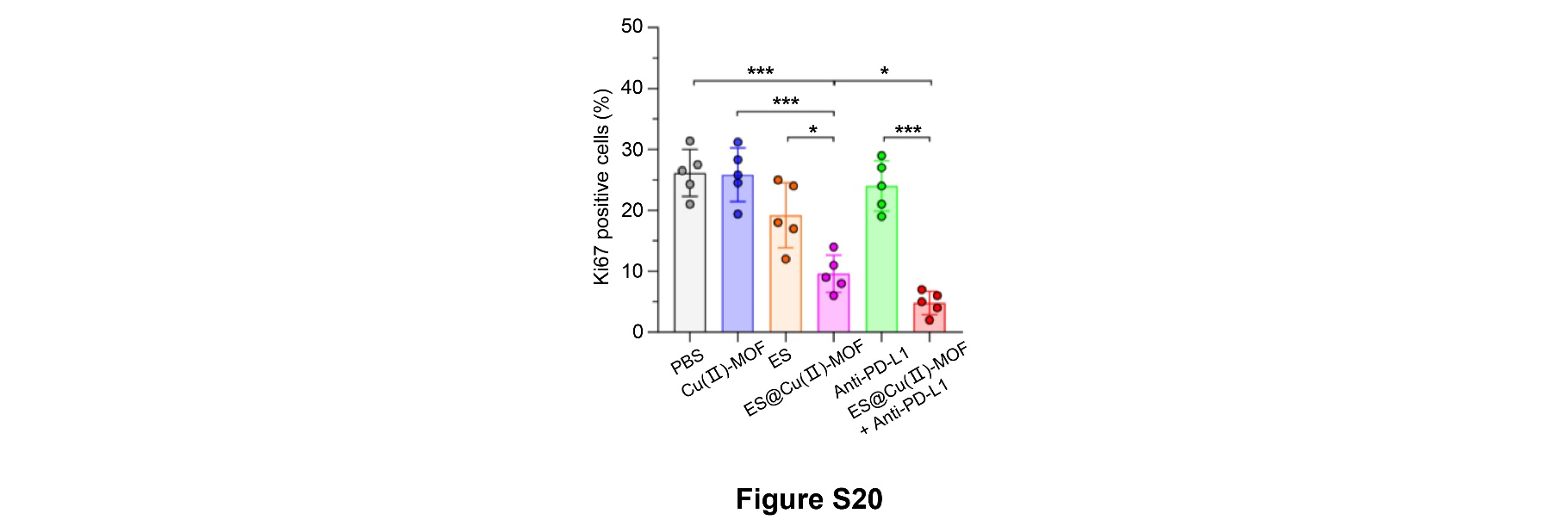


**Figure S20.** Quantification of Ki-67-positive cells in tumor tissues collected from 4T1 tumor-bearing mice following various treatments (n = 5). The data are presented as the means ± SDs; *p < 0.05; ***p < 0.001.


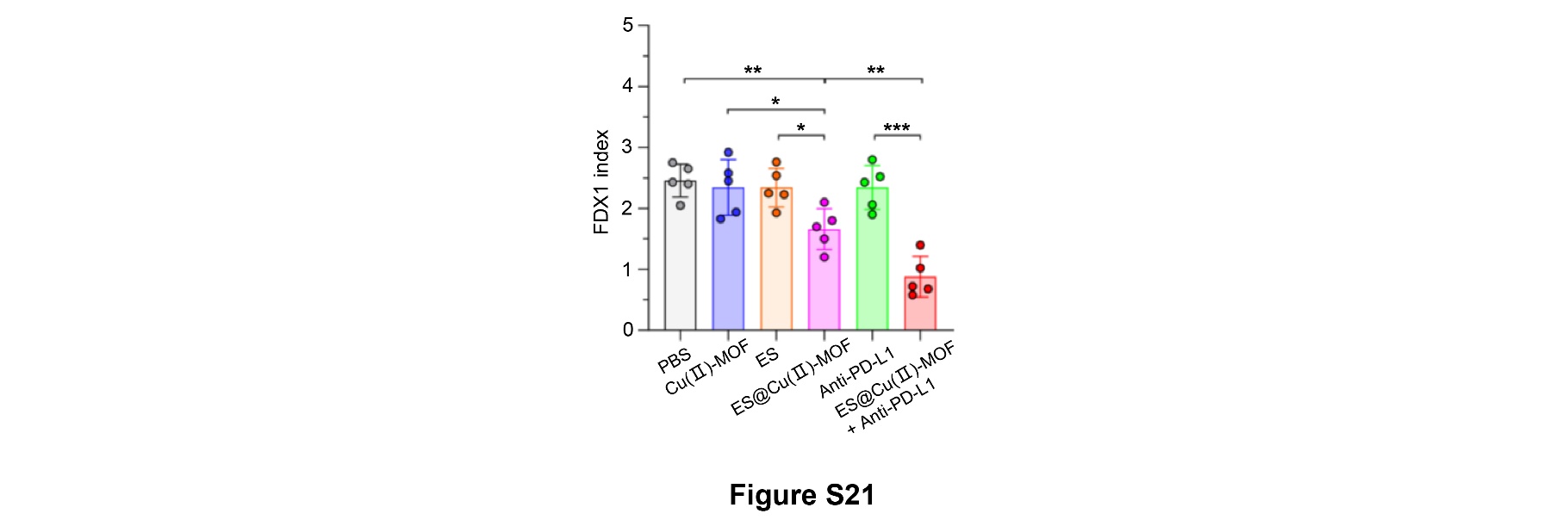


**Figure S21.** Quantification of FDX1-positive cells in tumor tissues collected from 4T1 tumor-bearing mice following various treatments (n = 5). The data are presented as the means ± SDs; *p < 0.05; **p < 0.01; ***p < 0.001.


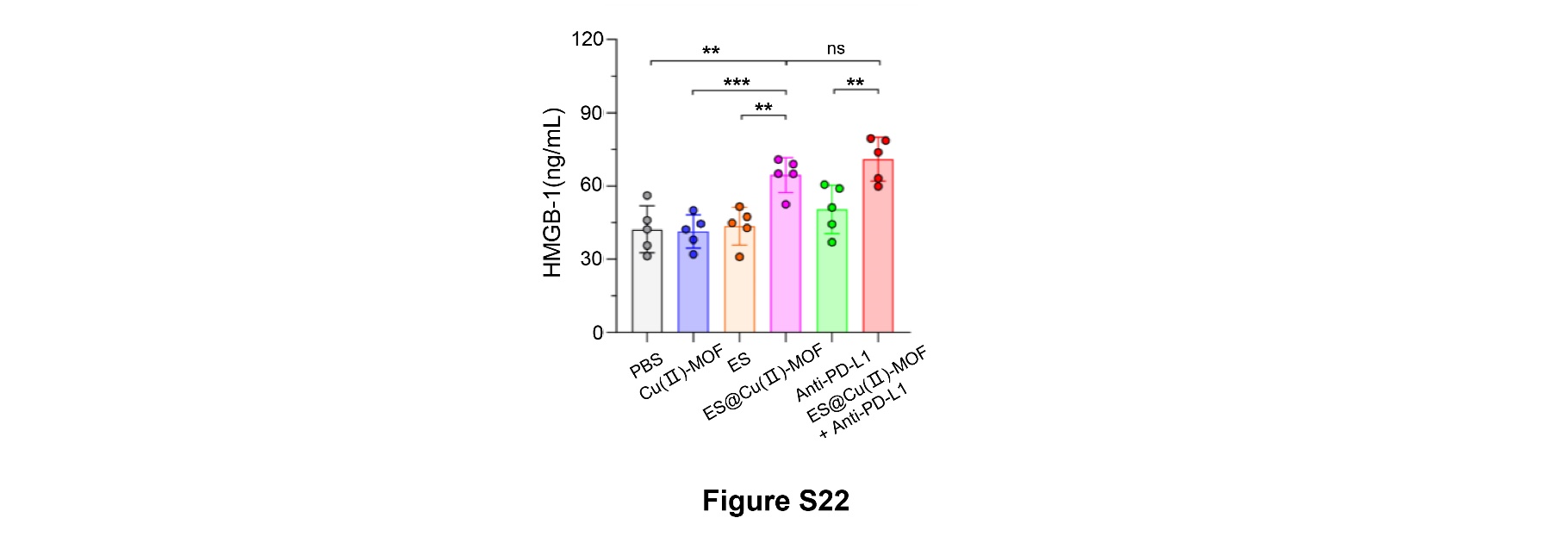


**Figure S22.** Serum HMGB-1 levels in 4T1 tumor-bearing mice following the indicated treatments (n = 5). The data are presented as the means ± SDs; ns, not significant; **p < 0.01; ***p < 0.001.


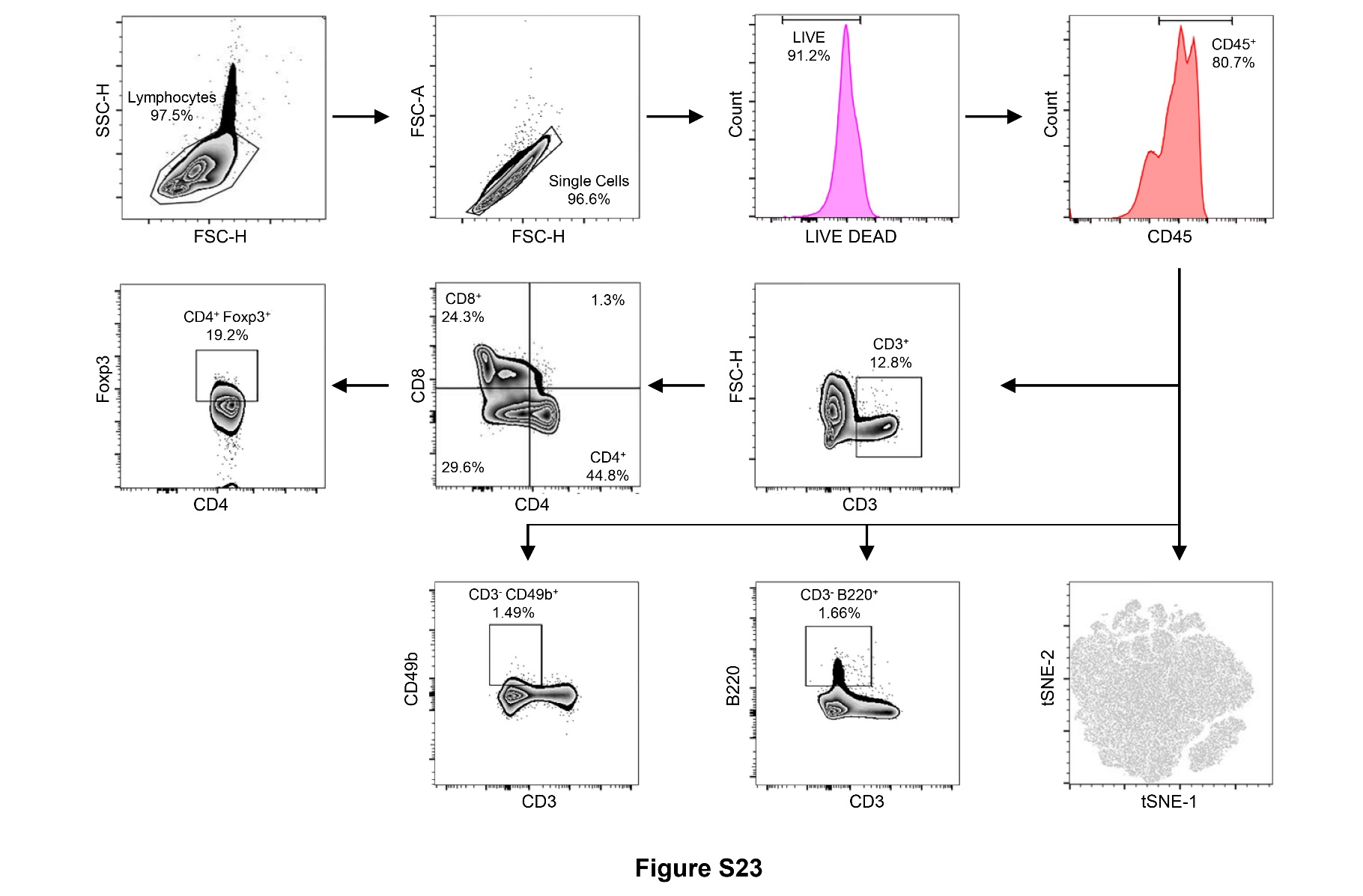


**Figure S23.** Gate strategy for identifying TILs in tumor tissue after various treatments.


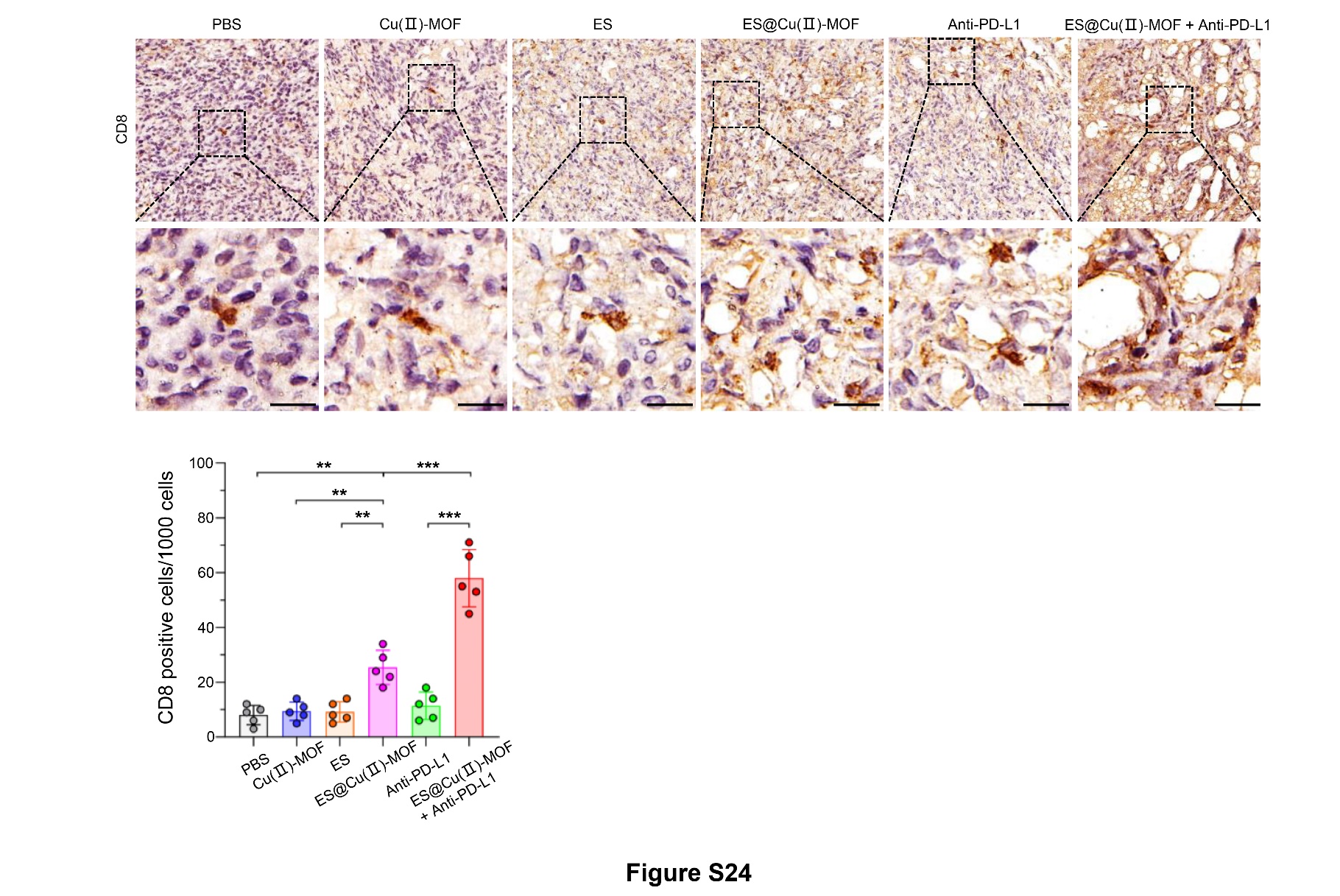


**Figure S24.** CD8 IHC staining of tumor tissues after ES@Cu(Ⅱ)-MOF + Anti-PD-L1 combination therapy (n = 5). Scale bar: 20 μm. The data are presented as the means ± SDs; **p < 0.01; ***p < 0.001.


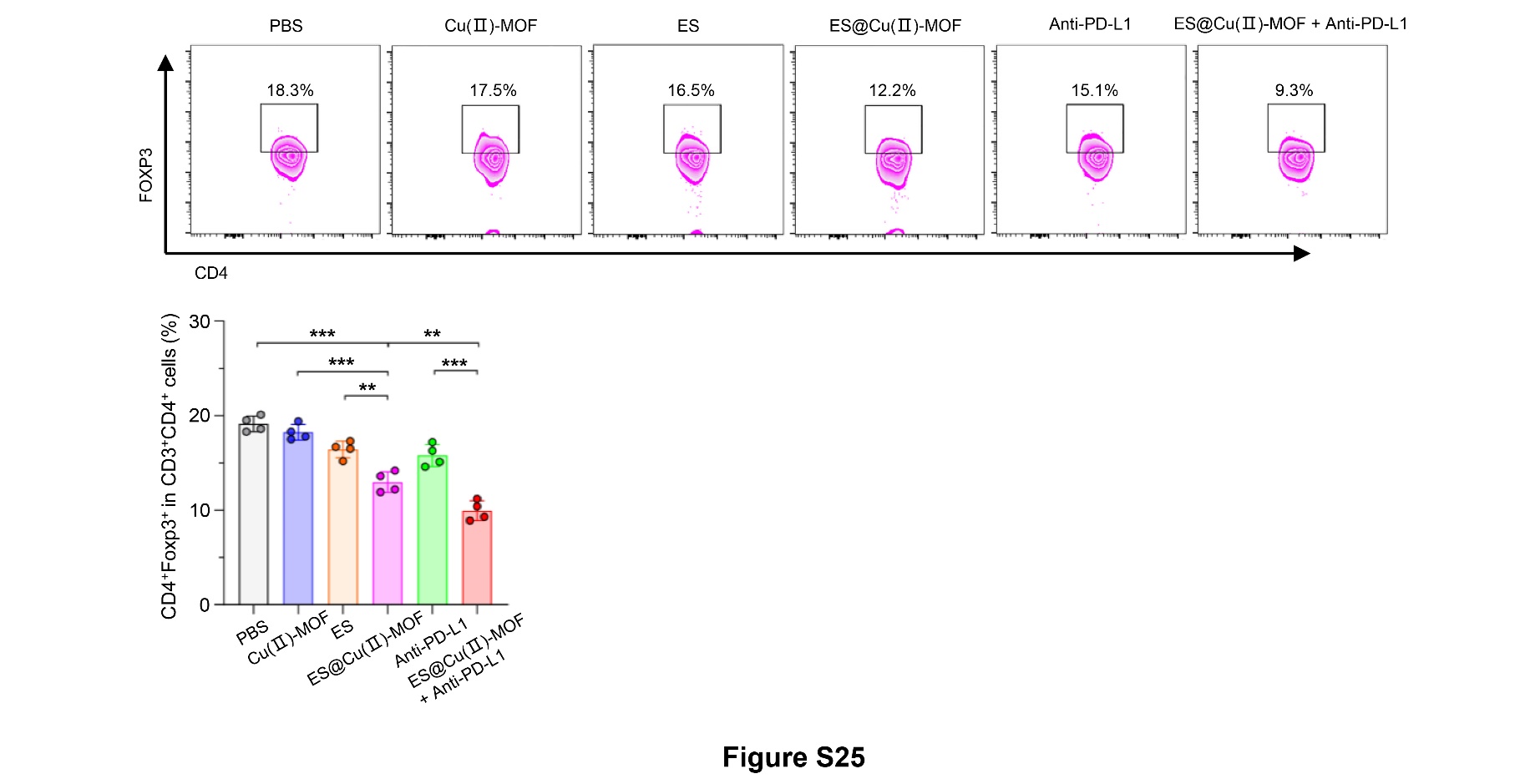


**Figure S25.** Flow cytometric analysis of CD4^+^Foxp3^+^ Tregs in tumor tissues from 4T1 tumor-bearing mice following the indicated treatments (n = 4). The data are presented as the means ± SDs; **p < 0.01; ***p < 0.001.


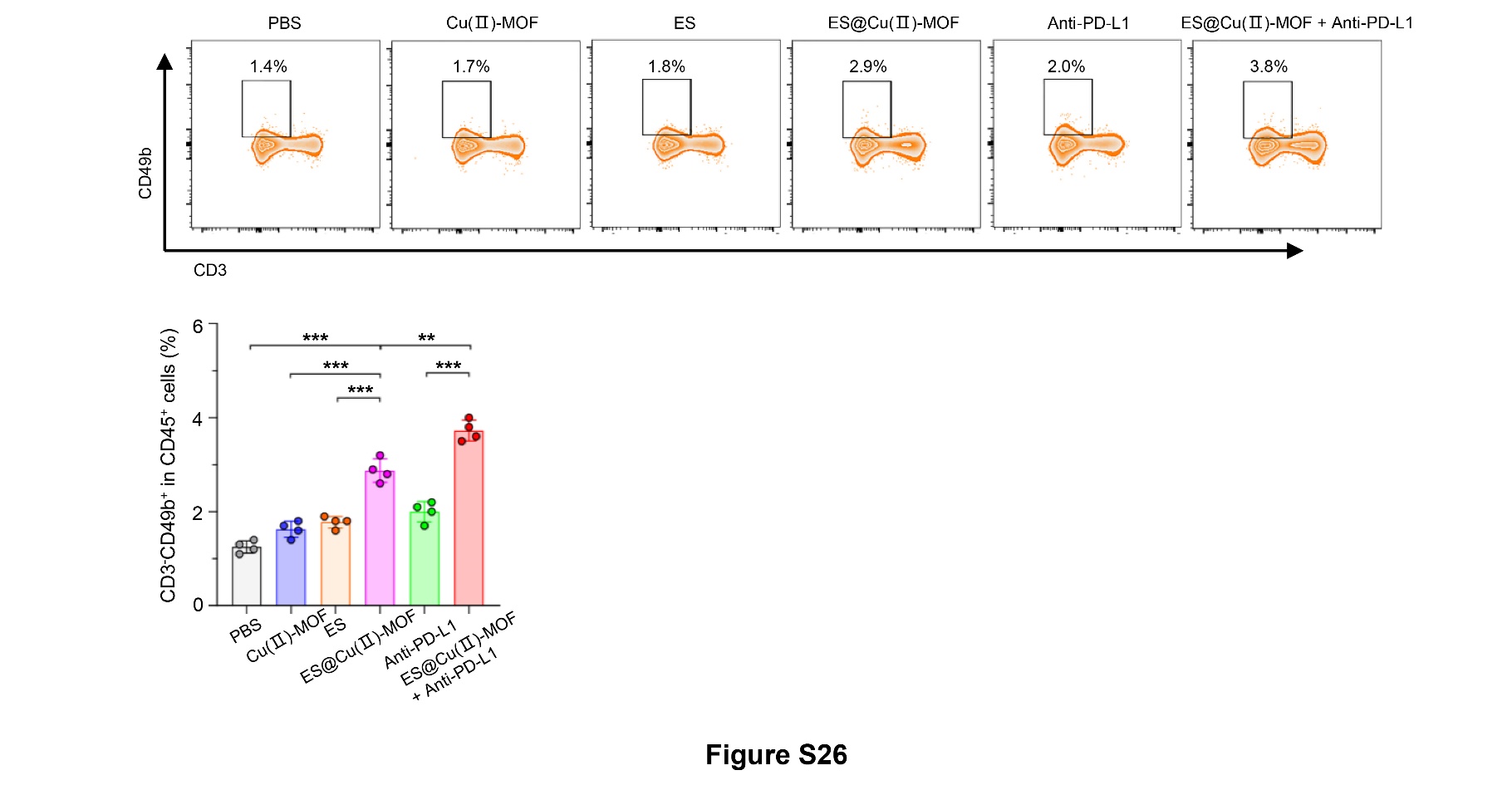


**Figure S26.** Flow cytometric analysis of CD3^-^CD49b^+^ NK cells in tumor tissues from 4T1 tumor-bearing mice following the indicated treatments (n = 4). The data are presented as the means ± SDs; **p < 0.01; ***p < 0.001.


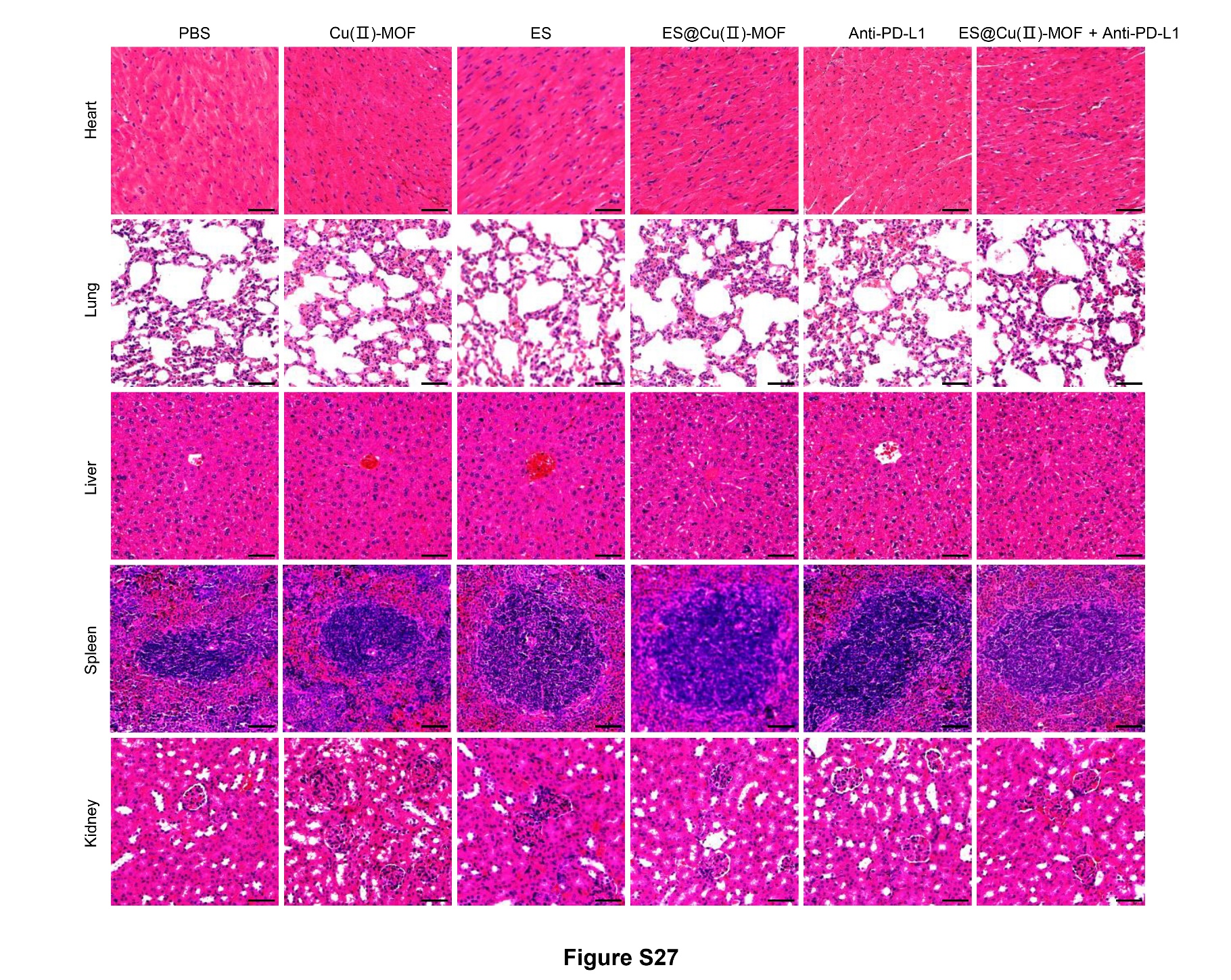


**Figure S27.** H&E images of major organs (heart, lung, liver, spleen and kidney) after ES@Cu(Ⅱ)-MOF + Anti-PD-L1 combination therapy. Scale bar: 50 μm.


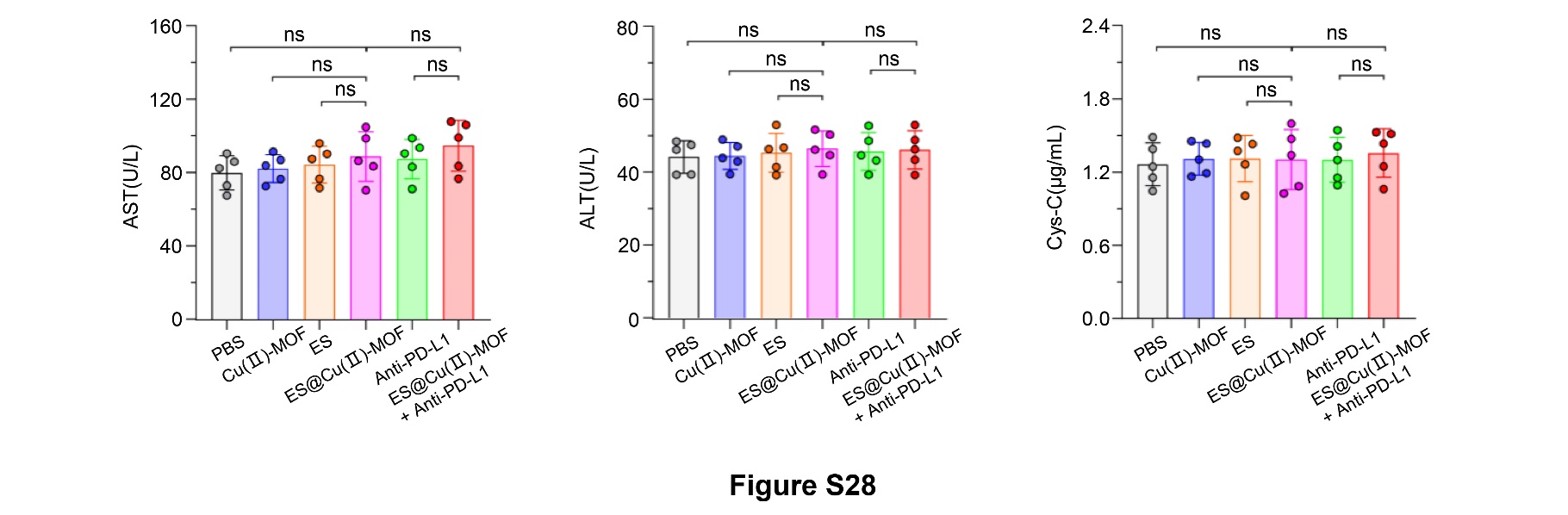


**Figure S28.** Serum biochemical analysis of liver function (AST and ALT) and kidney function (Cys-C) after ES@Cu(Ⅱ)-MOF + Anti-PD-L1 combination therapy (n = 5). The data are presented as the means ± SDs; ns, not significant.


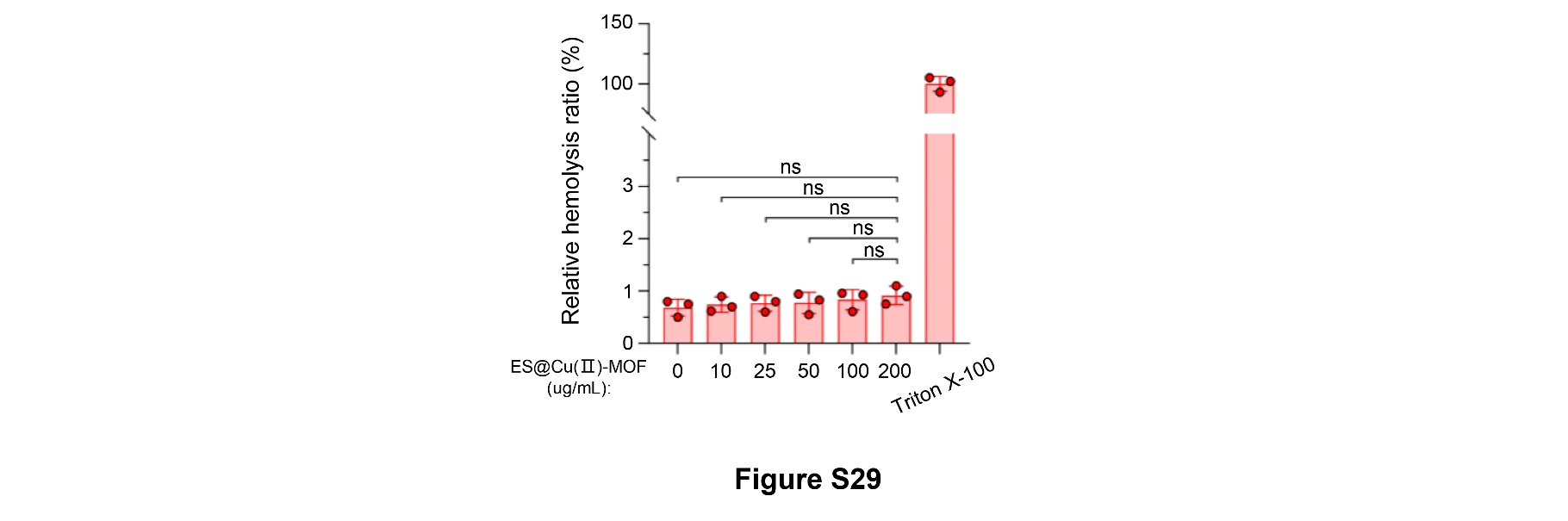


**Figure S29.** Hemolysis assay of ES@Cu(Ⅱ)-MOF NPs at different concentrations. Triton X-100 was used as the positive control, and 0.9% saline was used as the negative control (n = 3). The data are presented as the means ± SDs; ns, not significant.


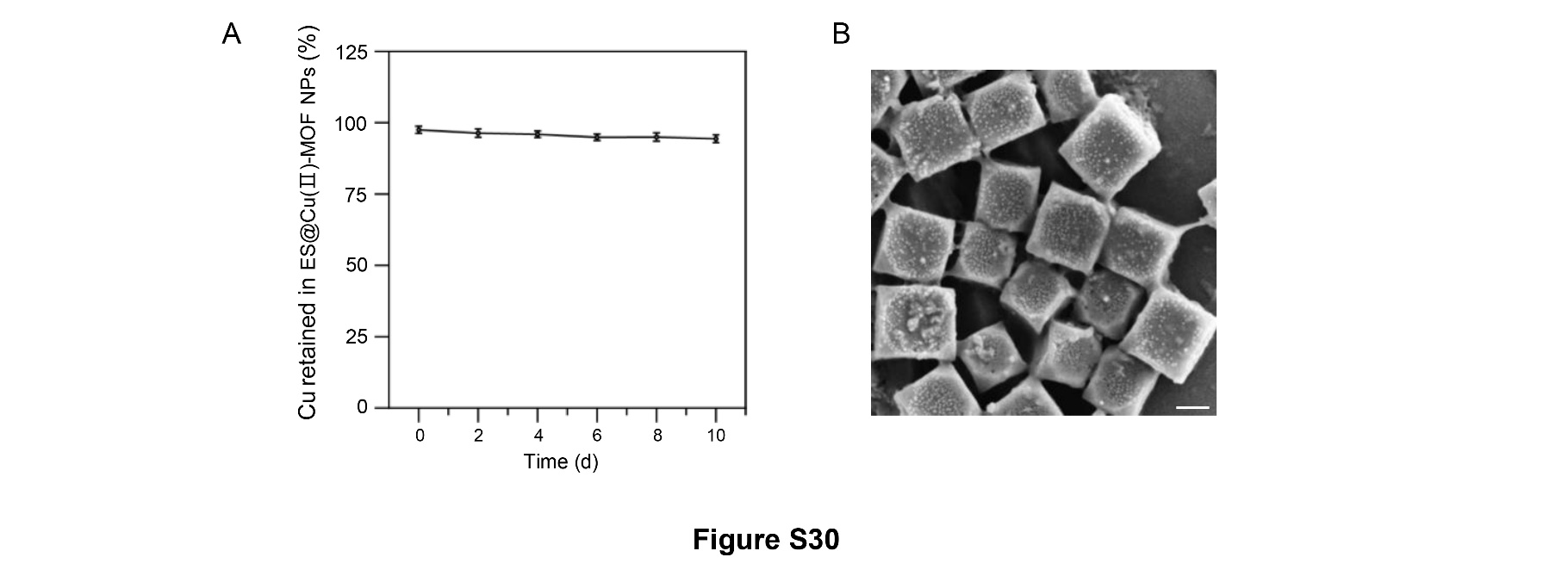


**Figure S30.** The stability of the ES@Cu(Ⅱ)-MOF NPs. ES@Cu(Ⅱ)-MOF NPs (1.0 mg/mL) were resuspended in 1 mL of DMEM containing 10% FBS. (A) After incubation for the indicated times, the amount of Cu retained in the solution was measured via ICP‒MS. (B) SEM images of the ES@Cu(Ⅱ)-MOF NPs after incubation in DMEM containing 10% FBS for 10 days. Scale bar = 100 nm.


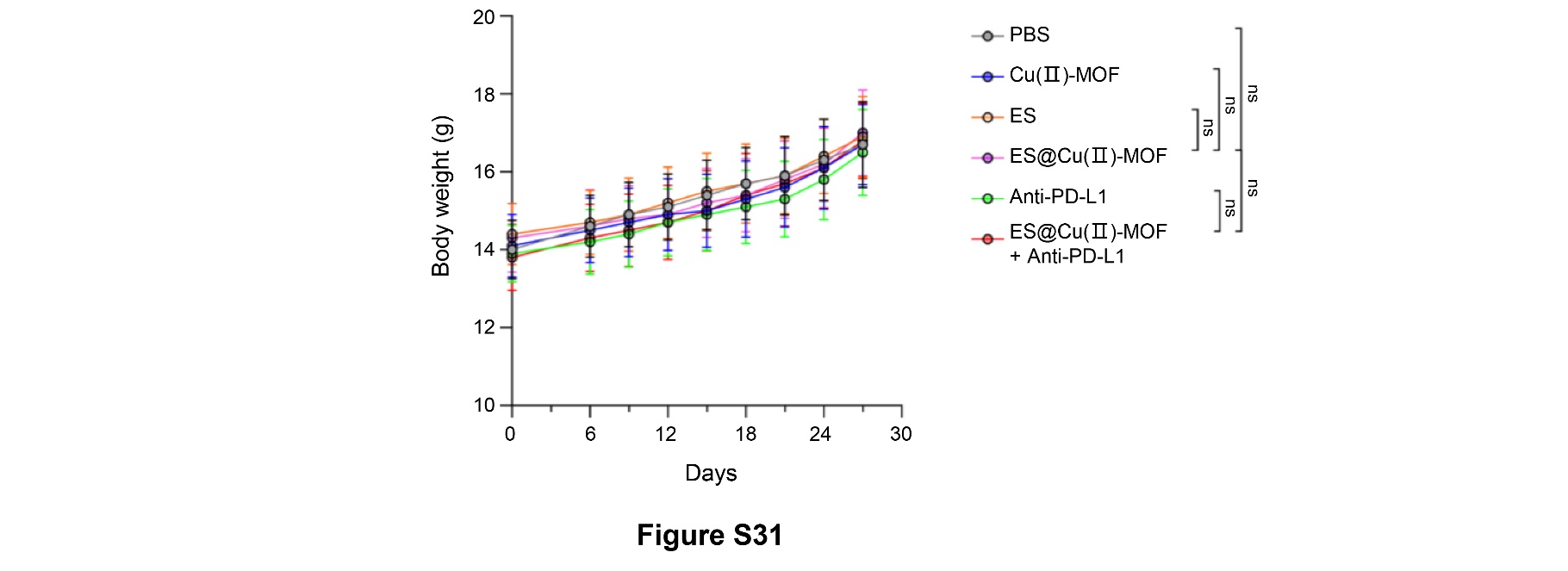


**Figure S31.** Body weights of the mice treated with ES@Cu(Ⅱ)-MOF + Anti-PD-L1 combination therapy (n = 5). The data are presented as the means ± SDs; ns, not significant.


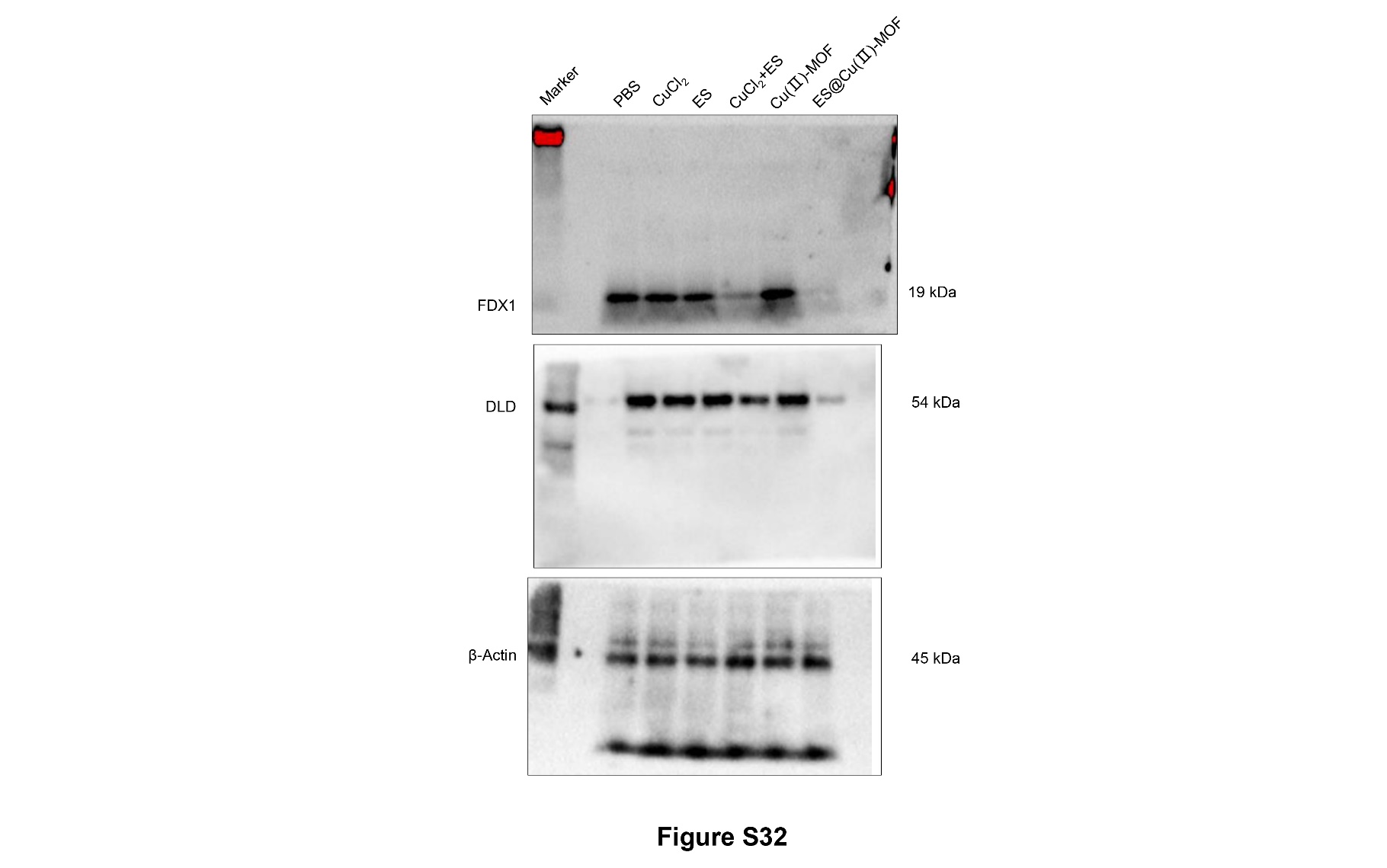


**Figure S32.** Uncropped western blot of figures.
